# Insomnia: the gut microbiome connection, prospects for probiotic and postbiotic therapies, and future directions

**DOI:** 10.1016/j.jare.2025.07.005

**Published:** 2025-07-10

**Authors:** Qiong Wu, Guangqi Gao, Lai-yu Kwok, Huimin Lv, Zhihong Sun

**Affiliations:** a Key Laboratory of Dairy Biotechnology and Engineering, Ministry of Education, Inner Mongolia Agricultural University, Hohhot, China; b Key Laboratory of Dairy Products Processing, Ministry of Agriculture and Rural Affairs, Inner Mongolia Agricultural University, Hohhot, China; c Inner Mongolia Key Laboratory of Dairy Biotechnology and Engineering, Inner Mongolia Agricultural University, Hohhot, China; d Collaborative Innovative Center for Lactic Acid Bacteria and Fermented Dairy Products, Ministry of Education, Inner Mongolia Agricultural University, Hohhot, China

**Keywords:** Insomnia, Probiotics, Postbiotics, Gut microbiota, Gut metabolome, Intervention trials

## Abstract

**Background:**

The gut microbiota is influenced by a complex interplay of factors including the host’s genetics, diet, and lifestyle. It plays a crucial role in regulating metabolic processes and is linked to various diseases. Growing evidence suggests that the gut-brain axis, which involves bidirectional communication between the gut microbiota and the brain, plays a crucial role in regulating sleep patterns and has significant implications in the development of sleep disorders.

**Aim of review:**

This comprehensive review aims to summarize the current knowledge on the mechanisms underlying insomnia and its association with the gut microbiota and metabolome. Probiotics and postbiotics are beneficial microorganisms and their components and metabolites, which offer potential in modulating the gut microbiota of the host and alleviating insomnia. We present an overview of recent progress in probiotic and postbiotic intervention trials for managing insomnia, highlighting both the potential benefits and limitations of existing research in this domain. Additionally, we offer suggestions for future directions in this field. Probiotics can regulate sleep-wake behavior by modulating gut microbiota and its metabolites, influencing endocrine, neuronal, and immune responses. In addition, to understand the role of probiotics in improving insomnia, a standardized sleep behavior index and sleep monitoring method are needed.

## Highlights

• Complex sleep monitoring and multifactorial triggers hinder insomnia research, necessitating regulatory factor reclassification.
• Insomnia is closely related to sleep regulatory factors, mitochondria, and intestinal flora and metabolite levels.
• Probiotics/postbiotics modulate sleep via gut-brain axis (vagal/immune/endocrine pathways) by targeting gut microbiota & metabolites.

## Graphical abstract

**Figure:**
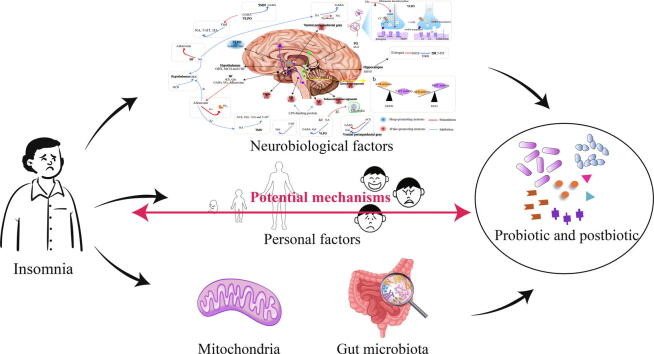

## Introduction

According to the Diagnostic and Statistical Manual of Mental Disorders, Fifth Edition (DSM-5), specific criteria must be met to diagnose insomnia, including: (a) Dissatisfaction with sleep duration or quality, including difficulty falling asleep, prolonged sleep, and early awakening, accompanied by associated symptoms. (b) The sleep disturbance causes clinically significant distress or impairment in social, occupational, educational, academic, behavioral or other important areas of functioning. (c) Insomnia episodes occur at least three nights per week, persisting for a minimum of three months. (d) Despite sufficient sleep opportunity, the sleep disturbance persists. (e) Insomnia cannot be attributed solely to another sleep-wake disorder or occur exclusively during the course of another sleep-wake disorder (e.g., narcolepsy, respiratory-related sleep disorders, circadian rhythm sleep-wake disorders, sleep disorders due to other medical conditions). (f) The sleep disturbance cannot be solely attributed to the physiological effects of a substance (e.g., substance use, medication). (g) The presence of coexisting mental disorders and physical conditions does not fully explain the complaints of insomnia. At present, insomnia and excessive daytime sleepiness are increasingly prevalent worldwide, posing significant challenges to public health. A cross-sectional study conducted during the 2011–2012 Health Survey on 54,722 European adults revealed a high prevalence of sleep problems, ranging from 16.6 % in Denmark and Italy to 31.2 % in Poland [1]. Sleep serves a critical role in repairing neuronal DNA damage incurred during wakefulness [2], making it imperative to understand the characteristics and underlying causes of insomnia for effective treatment strategies.

Insomnia is a complex disorder influenced by various factors, including genetics, neurobiology, endocrine, immune, and psychosocial factors [3]. Recent investigations into the gut microbiota have highlighted the role of gut dysbiosis in the development of neurological problems, including depression, anxiety, and insomnia [4], highlighting the significance of the gut-brain axis in health and disease. Consequently, regulating the gut microbiota has emerged as a potential avenue for managing these conditions. Probiotics and postbiotics have garnered attention as potential treatments for rebalancing the gut microecology and improving sleep quality.

In this comprehensive review, we conducted an extensive search of the PubMed, Web of Science, and Google Scholar databases using the keywords “probiotics”, “postbiotics” “gut microbiota”, and “insomnia”. Our primary objective was to explore the connection between the gut microbiota and insomnia. Furthermore, we present relevant clinical and animal intervention trials of probiotic and postbiotic interventions for insomnia to provide insights and suggestions for future research directions.

## Factors contributing to insomnia

### Neurobiological factors and their dysfunction

The disruption of neural pathways involved in regulating sleep-awakening, abnormalities in neurotransmitters and receptor function, and disturbances in hormone action are the primary mechanisms underlying insomnia. Gaining a comprehensive understanding of the neural circuits associated with various chemicals involved in sleep-wake transition is crucial for advancing strategies for managing insomnia.

#### Dysfunction of sleep regulatory factors (SRFs)

The brain contains numerous SRFs, predominantly neurotransmitters, but also including hormones and cytokines (summarized in Table 1). Some of these factors are sleep-promoting, such as γ-aminobutyric acid (GABA), galanin (Gal), adenosine, melanin concentration hormone (MCH), and melatonin (Mel). Conversely, there are wake-promoting factors, such as histamine (HA), acetylcholine (ACh), orexin (ORX), and noradrenaline (NA). Additionally, other factors, including 5-hydroxytryptamine (5-HT), dopamine (DA), and brain-derived neurotrophic factor (BDNF), are also involved in sleep regulation. Understanding the mechanisms and functions of SRFs and their receptors forms the foundation for comprehending the physiological role of these factors in the sleep-wake cycle (summarized in Table 1).

**Table 1:** Sleep regulation factors, receptors, primary sources, and findings.

Sleep regulation factors	Receptors	Primary sources	Findings
Melatonin (Mel)	MT_1_ MT_2_	Pineal gland	MT_1_ is associated with the regulation of circadian rhythms and primarily involved in the modulation of REM sleep, whereas selectively promoting NREM sleep [31]. MT_2_ increases non-rapid eye movement (NREM) sleep, while MT_1_ regulates rapid eye movement (REM) sleep. MT1 and MT2 receptors play opposite roles in mice, with MT1 knockout increasing NREM sleep and MT2 knockout decreasing it [31]. MT_1_ is periodically regulates 5-hydroxytryptamine (5-HT) activity in the dorsal raphe (DR) [32]. In rats, the activity of aralkylamine N-acetyltransferase (AANAT) in the pineal gland significantly increases by 100 times after the onset of darkness at night, and within a few minutes of light exposure, AANAT activity sharply decreases to daytime levels [33]. During the perimenopausal period, as estrogen secretion decreases, it disrupts the hypothalamic-pituitary-ovarian axis, leading to reduced Mel secretion and ultimately contributing to insomnia in perimenopausal women [34]. Mel has positive effects on neuroprotection in traumatic brain injury [35], [36], [37].
γ-aminobutyric acid (GABA)	GABA_A_ GABA_B_ GABA_C_	Ventrolateral Preoptic nucleus (VLPO)	GABA levels were found to be reduced by 30 % in patients with insomnia, potentially contributing to difficulties in initiating and maintaining sleep [38]. GABA can improve sleep, relieve mood, and stimulate brain activity [39,40]. GABA_A_ and GABA_C_ are involved in rapid inhibitory neurotransmission by regulating chloride ion flow [41]. GABA regulates the stress response by modulating the brain circuitry in the amygdala (Nuss, P., 2015). Activating GABAergic neurons in the VLPO projecting to the tuberomammillary nucleus (TMN) promotes slow wave sleep (SWS) [42,43]. The GABA_A_ agonist, diazepam, shows neuroprotective effects at low doses [44]. In contrast, injecting the GABA_A_ antagonist, bicuculline, into the TMN caused an increase in wakefulness [45]. Benzodiazepines are classical sleep aids that act by modulating GABA receptor complexes [46].
Glutamate	−	VLPO	Glutamate, the precursor of GABA, is produced through catalysis by glutamate decarboxylase.
Galanin (Gal)	Gal_1_ Gal_2_ Gal_3_	VLPO	The decrease of sleep duration observed in the elderly is closely related to Gal neuron function in the VLPO [47]. When the Gal neurons in the VLPO are damaged, the sleep homeostasis of mice is disrupted [48]. The Gal_1_R agonist M617 acts on the TMN to promote SWS, inhibiting arousal caused by histaminergic (HA) neuronal activity [49]. Gal neurons downstream to noradrenalegic neurons in the Locus Coeruleus (LC) and serotoninergic (5-HT) neurons in the DR [50], and Gal_1_R receptors are distributed in these regions [51]. Patients with Alzheimer's disease (AD) often experience sleep fragmentation, reduced total sleep time (TST) at night, daytime sleepiness, and circadian rhythm reversal, which are associated with the loss of Gal neurons in the VLPO [52]. The increase in Gal synthesis and release is the body’s compensatory or protection mechanism [48].
Adenosine	A_1_ A_2A_	Astrocytes, basal forebrain (BF)	Apoptosis of glutamatergic neurons in the BF results in decreased adenosine concentration during both awakening and sleep stage [53]. Adenosine inhibits the activity of arousal neurons, including orexin (ORX) neurons and HA neurons, by activating A_1_R in different hypothalamic regions to promote sleep [54,55]. A_2A_R regulates the sleep cycle. Its deficiency can attenuate the sleep-promoting effect of endogenous prostaglandin D_2_[56].
Prostaglandin D_2_	−	BF	Prostaglandin D_2_, a potent sleep-promoting substance, is primarily produced by leptomeninges in mice, with a dose and time-dependent effect [57].
Melanin-Concentrating Hormone (MCH)	MCHR_1_ MCHR_2_	Ventral periaqueductal grey matter, LC; Hypothalamus	Throughout the sleep-wake cycle, MCH neurons exhibit reciprocal discharge with ORX neurons [58], and MCHR_1_ gene is expressed in various cell types in the ventral tegmental zone, including glutamatergic, GABAergic, and dopaminergic cells [59]. MCH has the ability to acutely inhibit dopamine (DA) in the nucleus accumbens. By relieving the inhibition of local glutamate signal transduction, MCH helps restores the DA level [60]. In rats, the level of MCH in cerebrospinal fluid is higher during the day, while the level of ORX is higher at night when the rats were awake [61]. SD significantly reduces the number of active ORX and noradrenalegic neurons in the LC. However, the number of active MCH neurons does not decrease in REM sleep [13,14,23,62]. MCH neurons exhibit their highest firing rate during REM sleep [63].
Nitric oxide (NO)	−	BF, DR, hypothalamus	Systemic or intraventricular administration of nitric oxide synthase (NOS) inhibitors in rabbits and rats can reduce both NREM and REM sleep and increase wakefulness [64]. NOS neurons in the hypothalamus of mice promote NREM and REM sleep and contribute to long-term hypothermia [65]. Cholinergic neuron NO production in the BF promotes adenosine release [66,67]. The calcium/calmodulin-dependent protein kinase II signaling pathway in the DR helps regulate neuronal NOS activity. SD decreases calmodulin-dependent protein kinase II in the DR, thereby weakening the inhibitory effect on neuronal NOS and promoting sleep [68]. The presence of NO in the lateral hypothalamic area causes the loss of the wake-promoting OXA neurons, leading to sleepiness [23,69].
Noradrenaline (NA)	Noradrenergic β2 receptor	LC	REM sleep deficiency leads to increased NA levels in the brain, causing neuronal apoptosis and altered cell morphology. Treatment with prazosin restores NA levels and morphology [70]. Tyrosine hydroxylase microinjection in the LC region confirmed the role of NA in REM sleep, indicating that absence of REM sleep could cause mitochondrial damage [70]. Attenuating NA signaling reduces the likelihood of wakefulness induced by sound [71] During REM sleep, the level of NA in the brain increases. This elevated NA acts on α1 adrenergic receptors, leading to mitochondrial dysfunction, cytochrome *c* release, and activation of the intrinsic pathway of neuronal apoptosis induced by REM SD in rats [70]. Electrical stimulation of the LC and optogenetic activation of axons projecting to the VLPO modulates the activity of neurons in the preoptic area of the hypothalamus. Moreover, LC signals can be projected directly to the VLPO region [72].
Histamine (HA)	H_1_ H_2_ H_3_	TMN	H_1_ and H_2_ receptor activation can increase neuronal activity and discharge, causing long-term wakefulness and sleep suppression through H_1_R action on the TMN [73]. H_2_R activation affects cognitive activities in the awake state [74]. H_3_R mainly inhibits its own cell discharge, HA synthesis and its release through neuronal feedback regulation mechanism [19]. Intraperitoneal injection of ciproxifan, a selective H_3_R antagonist, could significantly increases the activity of TMN HA neurons and the HA concentration in the projection area, leading to arousal [75,76]. H_3_R is distributed in the cell bodies or dendrites of HA-dominated neurons, inhibiting the release of various neurotransmitters like acetylcholine (ACh), glutamate, NA, and 5-HT [77]. HA neurons often act in conjunction with other neurons and establish synaptic connections with NA neurons in LC and 5-HT neurons in DR, further promoting arousal by activating these neurons [78]. HA neurons in the TMN help regulate GABA and Gal pathways.
Acetylcholine (ACh)	nicotinic Ach (nACh) muscarinic (mACh)	Laterodorsal tegmental nucleus, pedunculopontine tegmental nucleus, BF	During REM sleep, frequent cholinergic projections and terminal activity in the SWS region are weak, indicating that increased ACh levels significantly reduce the occurrence of slow waves [79]. During REM sleep and wakefulness, the cholinergic input to the hippocampus is relatively high, but during NREM sleep, the ACh released by medial septum cholinergic neurons is significantly reduced [80]. Acetylcholinesterase inhibitors promote wakefulness and reduce the quality of NREM sleep by non-selectively enhancing cholinergic signal transduction [81]. nACHR knockout animals exhibit longer NREM and REM sleep, regardless of light conditions [82]. In Alzheimer's disease model mice, combined probiotic treatment beneficially enhances ACH secretion [83].
Orexin (ORX)	ORX_1_ ORX_2_	Hypothalamus	Chemical gene silencing of the ORX signal in the dorsal tegmental nucleus reduces REM sleep and interrupt REM sleep atonia [84]. Both ORX_1_ and ORX_2_ receptors help regulate sleep and wakefulness, but ORX_2_ plays a more important role in these processes and is a key receptor for regulating wakefulness and NREM sleep [85]. ORX neurons promote and stabilize wakefulness. They inhibit the effects of endogenous ORX neuropeptides by blocking ORX receptors [86], [87]. Highly selective ORX_2_ receptor antagonists induce sleep rapidly during early circadian rhythms, improve sleep quality, and do not affect wakefulness [88]. Glutamatergic synapses induce ORX-induced neuronal activation in the hypothalamus, implicating for insomnia treatment [89].
5-Hydroxytryptamine (5-HT)	5-HT_1_, 5-HT_2_ 5-HT_3_ 5-HT_4_ 5-HT_5_ 5-HT_6_ 5-HT_7_	DR	5-HT regulates sleep-wake behavior and suppresses REM sleep, and is modulated by hypothalamic ORX [90]. Abnormal 5-HT levels have been observed in the brains of individuals with insomnia [91,92]. Suppressing 5-HT in the DR reduces TST, decreases sleep depth, and increases the frequency of awakenings [93,94]. Impaired arousal in traumatic brain injury patients results in a 17 % and 29 % reduction in 5-HT neurons in the DR nucleus and NA neurons in the LC region, respectively [95]. The 5-HT level correlates positively with estrogen. Fluctuations in estrogen levels during perimenopause can lead to an imbalance in BDNF/ Tyrosine Kinase receptor B (TrkB)/5-HT2A signaling and weaken synaptic plasticity, ultimately resulting in mood disorders including insomnia [96]. *Ganoderma lucidum* extract promotes sleep by increasing 5-HT and the expression of *Tph2*, *Iptr3*, and *Gng13* genes in the hypothalamic 5-HT pathway that regulates sleep. [97]. *Tph2* knockout mice exhibit reduced sleep time [98]. 5-HT is a precursor for neurotransmitters like Mel and 5- hydroxyindoleacetic acid [99]. The main function of the 5-HT_1A_ receptor is to inhibit REM sleep [100].
Dopamine (DA)	D_1_R D_2_R D_3_R	Ventral periaqueductal grey, ventral tegmental area	Dopamine is a precursor for NA synthesis, DA neurons in the substantia nigra act on GABA neurons to regulate REM sleep [101]. There is a significant correlation between DA release in the cerebral cortex and the level of arousal [102]. Selective D_1_R agonists can increase wakefulness and shorten sleep duration [103]. D_2_R is involved in the mesolimbic reward pathway, regulating the switch from sleep to wakefulness and its maintenance. D_2_R agonists have a biphasic effect on sleep, that is, with low doses increasing sleep and high doses inducing wakefulness [103]. Dopamine acts on the basal lateral amygdala to express D_2_R, which contributes to the transition from NREM to REM sleep [104,105]. Deletion of the UBE3A gene reduces DA transporter function, which affects dopamine reuptake, leading to increased DA levels and wakefulness [106], and DA transporter knockout mice show significantly increased daytime arousal and significantly decreased NREM sleep [107].
Brain-Derived Neurotrophic Factor (BDNF)	TrkB p75	Hippocampus, prefrontal cortex and amygdala	Brain-derived neurotrophic factor interacts with 5-HT nervous system, regulating the survival and growth of neurons [108]. The blood BDNF level is an indicator of neural plasticity and can be used to evaluate the safety and effectiveness of probiotic intervention in improving stress, depression and sleep disorders [109]. TrkB.T1 knockout mice have normal NREM sleep time and homeostasis, but an increase in REM sleep time and decreases in sleep onset latency and sleep fragmentation [110]. Notably, BDNF can affect sleep homeostasis compared to individuals with healthy sleep patterns [111]. Serum BDNF levels in insomnia patients are significantly reduced, and the degree of reduction is significantly correlated with the severity of insomnia [112]. Estrogen receptor beta signaling reduces BDNF protein levels in the hippocampus, leading to impaired BDNF/TrkB/5-HT_2A_ signaling regulation, potentially causing insomnia in perimenopausal women [96].

Note. Sleep regulation factors mainly include some sleep-related neurotransmitters and hormones; Receptors means the receptors of sleep regulation factors; Primary sources are the specific brain regions generated and released by Sleep regulation factors.

#### Sleep-promoting factors

##### GABA is an important inhibitory neurotransmitter

Studies summarized in Table 1 provide insights into the role of GABA in promoting sleep, suggesting deficiencies in the secretion, release, and receptor-binding of GABA inhibitory neurotransmitters are associated with abnormal arousal, anxiety, and insomnia. Notably, approximately 80 % of GABA neurons in the ventrolateral preoptic nucleus (VLPO) co-expressed Gal and Gal receptors, including Gal_1_R, Gal_2_R, and Gal_3_R [5,6]. The Gal-Gal receptor system plays an important role in regulating sleep-wake patterns. Specifically, Gal_1_, promotes slow-wave sleep in the tuberomammillary nucleus (TMN) while inhibiting arousal caused by HA neuronal activity. Moreover, the downstream Gal-GABA neurons project to HA neurons in the TMN, NA neurons in the Locus Coeruleus (LC), and 5-HTergic neurons in the dorsal raphe (DR), exerting an impact on sleep regulation.

##### Mel is the earliest identified class of sleep-regulating factors

Mel exhibits a distinct circadian rhythm and mediates various physiological functions through Mel receptor 1 and Mel receptor 2 (Table 1). It has been found that the effect of sleep deprivation (SD) on gut barrier dysfunction may be stem from the inhibition of Mel rather than the mere absence of sleep itself [7]. Furthermore, a previous study demonstrated that cultured NA neurons could stimulate the PG to initiate Mel synthesis, but the production was transiently inhibited by TNF-α [8], indicate the involvement of inflammatory cytokines in the functioning of the PG, underscoring a significant association between insomnia and inflammatory responses.

##### Adenosine

Multiple studies have demonstrated that increased extracellular adenosine levels play a crucial role in enhancing total sleep time and slow wave activity [9,10]. As wakefulness duration extends, adenosine levels gradually rise in the brain and significantly decrease during sleep, aligning with the theory that the equilibrium of metabolites regulates sleep. Adenosine binding to A_1_ receptor inhibits ORX and HA neuronal activity, while binding to A_2A_ receptor inhibits prostaglandin D_2_ action, both contribute to sleep regulation [11,12]. In summary enzymes and receptors involved in adenosine production and metabolism jointly regulate the stability of adenosine levels, forming the adenosine signal system and maintaining internal physiological homeostasis.

##### MCH is a regulatory neuropeptide

MCH is primarily located in the neurons in the posterolateral and medial regions of the hypothalamus. Extensive research has confirmed that MCH plays a key role in the generation and maintenance of sleep, especially REM sleep. In rodents, its effect is exerted through a specific receptor, MCHR_1_, which is an essential regulator of homeostatic behaviors, including sleep and mood. MCH exhibiting a unique inhibitory effect on ORX and DA. This modulation of ORX and DA neuron output by MCH contributes to the improvement of sleep quality (Table 1).

#### Wake-promoting factors

##### NA is an important indicator for evaluating REM sleep

It was observed that plasma NA levels exhibit a significant increase in SD mice, correlating with the decrease in Mel levels [7]. Meanwhile, the heightened energy metabolic processes associated with extended wakefulness leads to oxidative damage, resulting in a significant reduction of 30 %–37 % in the number of NA neurons in the LC region [13,14]. Combined this information with the data presented in Table 1, we propose that NA is an important indicator for evaluating REM sleep, and that the VLPO serves as a key postsynaptic target for LC NA neurons in mediating wakefulness response. Additionally, a number of studies have shown that neural activity controls the dynamics of microglia through the noradrenergic β_2_ receptor signaling pathway, highlighting the dynamic transformation of microglia during the sleep-wake cycle and their role in sleep-related synaptic remodeling [15,16].

##### HA is important targets for improving sleep

About 80 % of HA neurons in the TMN express c-Fos during periods of arousal [17] and exerts its arousal effects through binding to the receptors H_1_R, H_2_R, and H_3_R. It should be noted that HA neurons can also affect the activity of other sleep regulatory systems (ACh, glutamate, NA, and 5-HT). Previous studies have demonstrated that *Limosilactobacillus reuteri* administration can convert L-histidine into HA in the intestine, which further enhances cAMP levels through H_2_R signaling pathway, thereby inhibiting downstream MEK/ERK MAPK signaling and suppressing colonic inflammation [18,19]. However, whether probiotics can improve sleep through a similar mechanism remains unknown.

##### Acetylcholine is a cholinergic neurotransmitter

Acetylcholine primarily functions by transmitting neural to the thalamus, neostriatum, BF and other regions. Its fiber tracts and ascending projections form an ascending reticular activation system [20], which affects sleep quality by impacting both NREM and REM sleep (Table 1). Cholinergic neurons play a crucial regulatory role by binding to ACh receptors. The presynaptic muscarinic ACh receptor modulates the release of various neurotransmitters, including GABA, glutamate, and ACh. Moreover, muscarinic ACh_1_R affects GABAergic transmission in VLPO neurons [21].

##### Orexin-A and Orexin-B

Orexin-A and orexin-B, also known as hypocretin 1 and hypocretin 2, are neurotransmitters secreted by neurons in the lateral and posterior hypothalamus to promote arousal [22]. It was found that SD for 12 h per day over a period of seven consecutive days could lead to a 24 % decrease in the number of ORX-containing neurons in the lateral hypothalamus in mice [23]. Thus, this reduction in ORX neurons may be the body’s compensatory mechanism to promote sleep. Studies have also confirmed that the ORX system directly innervates and excites NA, DA, 5-HT, HA, and ACh neurons, thereby promoting wakefulness (Table 1).

#### Other SRFs

##### [5-Hydroxytryptamine is a monoamine neurotransmitter

[5-Hydroxytryptamine is synthesized from the tryptophan through the action of tryptophan hydroxylases (peripheral TPH1 and central nervous system TPH2). Previous studies found that probiotics can regulate 5-HT levels by regulating tryptophan metabolism, potentially influencing the communication between gut microbiota and the sleep regulation system [24], [25], [26]. Interestingly, 5-HT has both arousal and hypnotic effects (Table 1). It is important to note that different subtypes of serotonin receptors in various brain regions have distinct pharmacological effects. Therefore, investigating the expression of different receptor subtypes is crucial for understanding the mechanisms involved. 4-Chloro-DL-phenylalanine (PCPA) can effectively block more than 95 % of 5-HT synthesis. Intraperitoneal injection of PCPA is thus commonly used to construct insomnia animal model for evaluate the effectiveness of interventions on improving insomnia associated with disruptions in the 5-HT system. Furthermore, 5-HT can potentially impact sleep by influencing Mel production. In summary, current research suggests that 5-HT is recognized as having a major function in the regulation of sleep-wake behavior and the suppression of REM sleep.

###### DA

The primary source of DA in the brain is the ventral tegmental area and the substantia nigra pars compacta. Since DA regulates the interaction between wakefulness or sleep by targeting different downstream receptors, maintaining the stability of DA levels and the corresponding receptors is essential for proper functioning of the sleep-wake system (Table 1).

#### BDNF was proposed as a potential biomarker for clinical diagnosis of insomnia

BDNF is expressed in the brain, including the cerebral cortex, olfactory bulb, BF, midbrain, and hypothalamus. The specific receptor for BDNF is TrkB [27]. Activation of TrkB triggers multiple signaling cascades, such as phospholipase C gamma, extracellular signal-regulated kinase (Erk), and protein kinase B pathways, which promote neuronal synaptogenesis and neurogenesis, thereby increasing the overall brain plasticity [28]. Numerous studies conducted in the past decade have elucidated the significant role of BDNF in individuals with sleep structure disorders and insomnia. Subsequently, BDNF was proposed as a potential biomarker for clinical diagnosis of insomnia [29]. In addition, BDNF function and regulation are also influenced by estrogen receptor beta. Table 1 summarizes current evidence of the role of TrkB and BDNF in mammalian and human sleep, establishing a connection between BDNF receptors, sleep abnormalities, and mental illness.

#### Nitric oxide

Nitric Oxide (NO) is a highly lipophilic gas through the catalysis of arginine by NO synthase. In the sleep-wake process, NO exerts its effects by post-translational modification of specific amino acid residues or cysteine *S*-nitrosylation in proteins. This modification inhibits the activity of pro-arousal neurons, including adenosine, DR, 5-HT neurons, and ORX neurons. Conversely, other studies, such as Dzoljic et al., have reported contradictory findings, where NO synthase inhibitors could increase sleep and reduce wakefulness in rats [30]. Therefore, further investigation is needed to fully understand the role of NO in sleep regulation.

The available evidence from current studies on sleep regulation and neuropharmacology strongly suggests that sleep regulatory systems hold promise as targets for treating insomnia. It is important to note that these systems do not operate in isolation; rather, they exert their effects by modulating various brain nuclei or neurons to facilitate the switch between wakefulness and sleep.

#### Dysfunction of sleep-wake neuron interactions

**Fig. 1:**
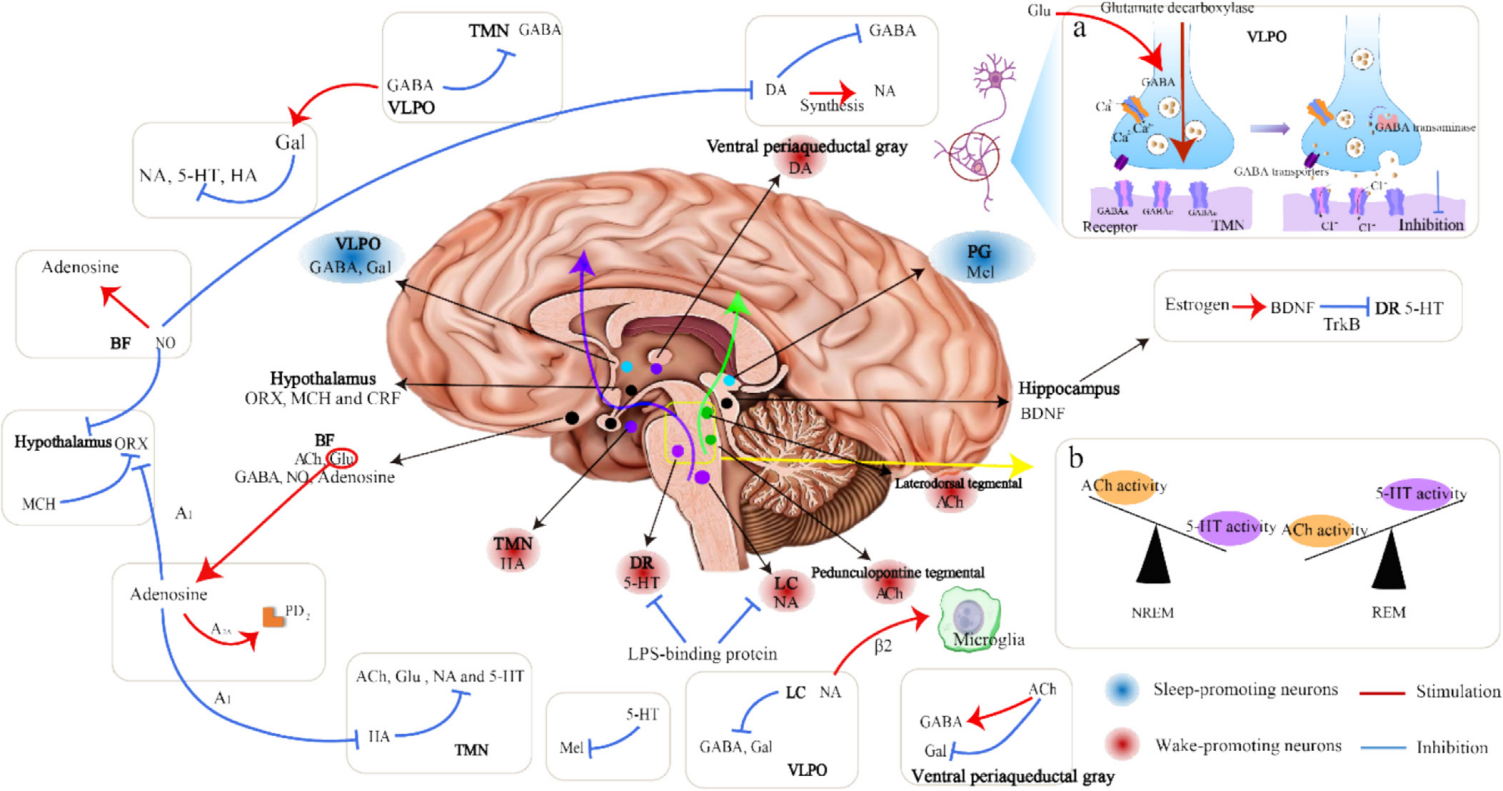
Sleep regulatory factors and their mechanisms of sleep-wake regulation. The figure shows the brain regions where various sleep regulatory factors are released and the connections between the different regulators. Two types of sleep regulatory neurons are depicted: sleep-promoting neurons (illustrated with blue circles) and wake-promoting neurons (illustrated with red circles). The sleep-promoting center primarily inhibits cortical activity through the release of GABA and Gal in the Ventrolateral Preoptic Nucleus (VLPO). Additionally, melatonin (Mel) released from the pineal gland of the brain exerts a sleep-promoting effect. There are two wake-promoting pathways (represented by purple and green arrows, respectively), the neurons and brain regions involved in the pathway are indicated by the dots in purple and green. Other sleep regulatory factors (illustrated with black circles) have an influence on the regulation of the sleep-wake system. (a) The activation process of neurotransmitter transmission at the synapse is illustrated, using GABA as an example. GABA is synthesized, stored, and transported in vesicles through vesicular inhibitory amino acid transporters until during neurotransmission from the presynaptic membrane of the preceding neuron into the synaptic cleft, where it binds to corresponding receptors (GABA_A_R and GABA_C_R) on the postsynaptic neuron to open the ion channels, allowing Cl^-^ to flow into cell, thereby reducing the likelihood of generating action potentials and inhibiting the activity of the next neuron. GABA_B_R metabotropic receptors can either open K^+^ or Ca^2+^ channels through G protein activation, removing positively charged ions from cells, or hyperpolarize cell membranes, leading to neuronal inhibition and promoting sleep. GABA can be transported back to the presynaptic membrane neurons by GABA transporters from the synaptic cleft for reuse, and it may also be inactivated by GABA transaminase. (b) The balance between the ACh and 5-HT systems in the brainstem region regulates NREM and REM sleep. Abbreviations: Sleep regulatory factors: 5-HT, 5-hydroxytryptamine; Ach, acetylcholine; BDNF, brain-derived neurotrophic factor; DA, dopamine; GABA, γ-aminobutyric acid; Gal, galanin; HA, histamine; Mel, melatonin; MCH, melanin concentration hormone; NO, Nitric oxide; NA, noradrenaline; ORX, orexin. Brain location (bolded font in the figure): BF, basal forebrain; DR, dorsal raphe; LC, locus coeruleus; TMN, tuberomammillary nucleus; VLPO, ventrolateral preoptic nucleus. Receptor: β2, noradrenergic β2, receptor; GABA receptor including GABA_A_, GABA_B_ and GABA_C_; TrkB, tyrosine Kinase receptor B. NREM, non-rapid eye movement; REM, rapid eye movement.

A key mechanism in normal sleep-wake regulation is the reciprocal inhibition between the VLPO and monoaminergic cell populations, including neurons in the LC, TMN, and DR (Fig. 1). The VLPO serves as the sleep-promoting center, while the LC, TMN, and DR are involved in promoting wakefulness. The GABAergic transmission actively inhibits the wake-promoting centers within the hypothalamus and brainstem, effectively reducing their activity. Moreover, GABA receptors in the VLPO that project to the TMN contribute to sleep promotion. At the same time, GABAergic neurons co-express Gal, and their somas and dendrites extend to the TMN, forming synaptic connections. These GABA/Gal neurons also project to NA neurons in the LC and 5-HT neurons in the DR, further facilitating sleep. Adenosine, inhibits the activity of wake-promoting neurons, such as ORX neurons and HA neurons. Therefore, the BF utilizes GABA and adenosine to enhance inhibitory influences on wake-promoting regions.

There are two main components of the ascending reticular activation system that contribute to awakening (Fig. 1). 1) Involves NA neurons in the LC, 5-HT neurons in the DR, HA neurons in the TMN, and DA neurons in the ventral periaqueductal gray. The HA neurons in the TMN form synaptic connections with NA neurons in the LC and 5-HT neurons in the DR, initiating cerebral cortex activity and promoting awakening. The NA neurons in the LC directly inhibit sleep-related neurons in the hypothalamic preoptic area and VLPO, further supporting wakefulness. Moreover, ORX-A/B and MCH-containing lateral hypothalamic neurons and ACh-containing BF neurons contribute to the wakefulness pathway in the brainstem. These neurons enhance the activation of cholinergic neurons in the BF and cortical regions, promoting wakefulness. 2) Involves cholinergic neurons located in the pedunculopontine tegmental and laterodorsal tegmental. These neurons initiate cortical activity by activating thalamus relay neurons. Additionally, afferent signals from neurons in the monoamine system, such as NA and 5-HT, directly inhibits VLPO neurons. The sleep-promoting and wake-prosmoting brain regions interact to modulate neuronal activity, resulting in distinct circadian rhythms and a normal sleep-wake state. However, a simultaneous disorder or dysfunction occurs in either of these systems may lead to insomnia.

#### Dysfunction of cholinergic and amine systems

Experiments conducted in cats found that manipulating the cholinergic (ACh) and amine (5-HT) systems in the brainstem can change the structure of sleep [113]. These two systems plays a crucial role in determining the cycles of NREM and REM sleep (Fig. 1b). During healthy sleep, the amine system is active during NREM sleep and becomes inactive at the onset of REM sleep [114]. Moreover, through cholinergic/anticholinergic stimulation experiments conducted on individuals with normal sleep, the hypothesis of an interactive model between NREM sleep and REM sleep has been validated, and it has been proposed that cholinergic stimulation can advance the occurrence of REM sleep episodes in both animals and humans.

#### Dysfunction of the PG

**Fig. 2:**
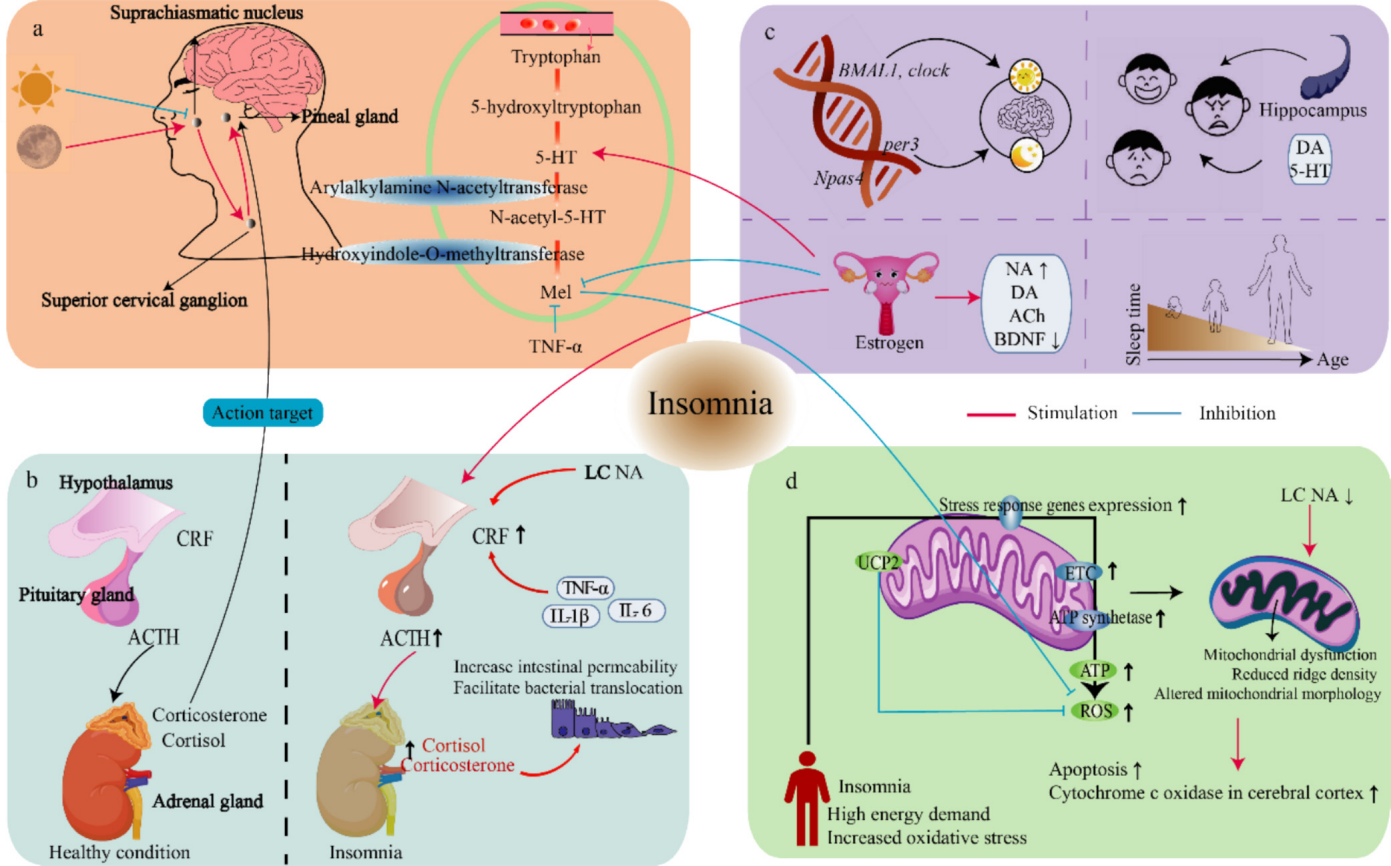
Other sleep regulatory mechanisms. (a) The pineal gland (PG) region usually regulates Mel synthesis in response to light signals. Damage to the PG can disrupt the levels of Mel and 5-HT and their metabolism. In darkness, axons from suprachiasmatic nucleus nerve cells release signals that act on the paraventricular nucleus of the hypothalamus. The paraventricular nucleus cells transmit the signal to the superior cervical ganglion, which then transmits the signal to the pinealocytes. In addition, Mel production in the PG is transiently inhibited by the TNF-α, while the PG is also a target for corticosterone action. (b) The hypothalamic–pituitary–adrenal (HPA) axis is the primary stress response system. Insomnia can lead to increased cortisol/corticosterone levels in the HPA axis, which may be associated with NA in LC neurons and some pro-inflammatory factors (including TNF-α; IL-1β; IL-6), and affect intestinal barrier function. (c) Insomnia exhibits individual variations. Genetic factors include clock genes, which regulate circadian rhythms, and genes regulating certain sleep regulators; and gender-related estrogens, which affects the HPA axis and regulates including DA, Mel, NA, BDNF and 5-HT. In additions, age and mood paly significant roles in sleep patterns, mainly related to DA and 5-HT. As individuals age, the physiological function of the body gradually declines, and the PG may undergo atrophy and eventually calcification, leading to a weakening or disappearance of rhythm of the biological clock. (d) Mitochondria provide energy to the cell through oxidative phosphorylation, a process that involves electron transfer and proton gradient formation. Electron transfer occurs between the electron transport chain (ETC) and the four complexes of ATP synthase. Insomnia often results in the accumulation of ROS, elevated expression of stress response genes (include antioxidants gens (SOD1, GSTS1, GSTO1, catalase) and Mitochondrial stress genes), disruption of energy balance and excessive oxidative stress accumulation can impair mitochondrial function and alter the morphology of mitochondria. However, whether these alterations contribute directly to insomnia remains to be clarified. Furthermore, downregulation of NA in LC neurons causes mitochondrial damage. Abbreviations: 5-HT, 5-hydroxytryptamine; Ach, acetylcholine; ACTH, adrenocorticotropic hormone; BDNF brain-derived neurotrophic factor; CRF, corticotropin-releasing factor; DA, dopamine; ETC, electron transport chain; IL, means interleukin; LC, locus coeruleus; Mel, melatonin; NA, noradrenaline; ROS, means reactive oxygen species; TNF, means tumor necrosis factor; UCP2, means mitochondrial uncoupling protein 2.

The PG is located in the posterior part of the cranial fossa and serves as the site for the synthesis and release of Mel (Fig. 2a). A previous study showed that pinealectomy could eliminate many seasonal responses [114]. Prolonged coffee intake has been associated with a reduction in PG parenchyma, potentially impacting sleep quality later in life [115], a decrease in PG volume may be related to REM sleep behavior disorder [116]. To further investigate the relationship between PG parenchymal volume, nighttime total sleep time, sleep quality index, and Mel levels, cranial magnetic resonance imaging was performed on 30 women with fibromyalgia. The findings revealed a significant correlation between PG volume and total sleep time, as well as nighttime Mel levels [117]. Therefore, the loss or changes in PG volume can lead to a range of sleep-related issues. A recent study has observed that sleep disturbances in heart patients were due to inflammatory macrophage recruitment, superior cervical ganglia fibrosis, leading to degeneration of their innervation of the PG and reduced Mel release [118]. However, another study reported unchanged PSG-monitored sleep following pinealectomy [119]. These reports highlight the existing controversy surrounding the effects of the PG on sleep.

#### Hypothalamic-pituitary-adrenal (HPA) axis overreaction

The HPA axis is the major endocrine pathway involved in regulating stress responses, and cortisol and corticosterone are the final products (Fig. 2b). Animal studies have demonstrated that SD mice experience an increase of in salivary corticosterone levels [120]. Similarly, in healthy individuals, sleep disorders have been found to enhance HPA axis reactivity [120]. Moreover, accumulating evidence supports the view that HPA axis activity is enhanced in individuals with insomnia exhibit excessive arousal [121]. Consequently, the excessive production of cortisol resulting from the overactivation of the HPA axis can stimulate wakefulness, disrupt circadian rhythm, and exacerbate insomnia symptoms. As mentioned above, in male subjects, poor sleep quality has been associated with a significant increase in cortisol stress response, while this effect was not observed in female subjects. Hence, the influence of HPA axis response appears to be gender-dependent [122]. This may be due to the interaction between estrogen and the HPA axis (see below for details).

Extensive evidence supports the interaction between cortisol and immune cells, as well as its control over cytokine release. Elevated levels of pro-inflammatory cytokines stimulate the secretion of corticotropin-releasing factor, which subsequently triggers the release of a large amount of cortisol from the adrenal glands to inhibit inflammation. Additionally, the HPA axis and heightened inflammatory response can disrupt the integrity of the gut barrier, increase intestinal permeability, and facilitate bacterial translocation and immune cell infiltration, resulting in a ‘leaky intestine’. The escape of toxic metabolites into the bloodstream contributes to the activation of the HPA axis, leading to increased cortisol production [123].

#### Personal factors

Studies have consistently demonstrated a strong genetic influence on insomnia. Candidate gene studies in animals and humans have identified associations between insomnia phenotypes and circadian clock genes (Fig. 2c), including *BMAL1/mop3* [124], *per* [125], *clock* [126], and *Npas4* [127]. Furthermore, a 44-base pair insertion/deletion polymorphism in the 5′ regulatory region of 5-HT transporter gene is associated with primary insomnia [128]. As individuals age, the physiological function of the body gradually declines, and neuronal decline or loss occurs in the aging brain [52]. At the same times, sleep problems also exhibit sex differences, with difficulties in initiating and maintaining sleep being more prevalent in women. One potential explanation for the gender disparity in insomnia is the influence of estrogen, which enhances HPA axis activity, increases 5-HT levels, and modulates neurotransmitters, including DA, Mel, endorphins and 5-HT [34,96,129]. It is worth noting that 5-HT and other neurotransmitters like DA are produced in the intestines, surpassing the amount produced in the brain and at a faster pace [130]. These neurotransmitters, produced by gut neurons, have the potential to improve mental well-being, enhance happiness, and even boost memory.

The aforementioned signaling pathways and regulatory systems contribute significantly to the mediation of insomnia. However, it is important to note that insomnia is a multifaceted condition involving various complex physiological processes. These include disruptions in the levels of different SRFs, brain dysfunction resulting from traumatic brain injuries, the influence of specific regulator factors on the neuronal cell populations involved in sleep homeostasis and various personal characteristics are increasingly recognized as potential influences on insomnia.

### Mitochondria and insomnia

Mitochondria are responsible for synthesizing ATP, the primary energy source for cells through oxidative phosphorylation. Even brief disruptions in ATP supply can have a major impact on synaptic activity. In a rat study, it was observed that compared to the sleep state, mRNA expression levels of cytochrome *c* oxidase subunit I, NADH dehydrogenase subunit 2, and 12S rRNA in cerebral cortex mitochondria were higher after 3 hours of wakefulness and the dynamics of night shift workers and the expression of rate-limiting mitochondrial enzymes exhibit circadian oscillations, indicating heightened mitochondrial activity during wakefulness [131,132]. During wakefulness, energy consumption increases, necessitating ATP production by mitochondria to fulfill the body’s energy requirements. This process involves electron transfer and the establishment of a proton gradient across the inner mitochondrial membrane through the electron transport chain and ATP synthase complexes. Electron leakage from the electron transport chain can generate reactive oxygen species and free radicals. During sleep, the brain utilizes reactive oxygen species accumulated during wakefulness, and the antioxidative Mel help inhibits reactive oxygen species. In addition, mitochondrial uncoupling protein 2, located in the mitochondrial inner membrane, acts as a buffer for excessive reactive oxygen species production during SD, ensuring normal mitochondrial function [133]. With the onset of SD, there is an elevated accumulation of reactive oxygen species, increased oxidative stress, and subsequent mitochondrial dysfunction, leading to apoptosis (Fig. 2d). This highlights the importance of maintaining a balanced mitochondrial function and minimizing oxidative stress for proper cellular functioning and overall health.

Interestingly, reduced sleep has been found to impact mitochondrial morphology. In mice, a decrease in spinal density was observed in the frontal cortex after four hours of sleep reduction. This was accompanied by the extension of mitochondrial outer membrane and expansion of the intermembrane space, leading to vacuole formation [134,135]. However, it is important to note that if sleep is recovered within a certain period of time, normal functional morphology can be restored. At present, there is convincing evidence suggesting that insomnia can affect mitochondrial function and morphology. However, there is a lack of definitive studies confirming whether disruptions in mitochondrial morphology can directly result in insomnia disorders.

In summary, the factors that regulate sleep are complex and varied, and multiple factors individually or collectively regulate sleep; therefore, when using probiotics as a means to regulate insomnia, we usually need to understand the cause of insomnia in more detail and demonstrate the regulatory mechanism of the probiotic by identifying specific pathways.

### Alterations of gut microbiota, gut microbiota-derived metabolites, immune and other factors in insomnia

**Fig. 3:**
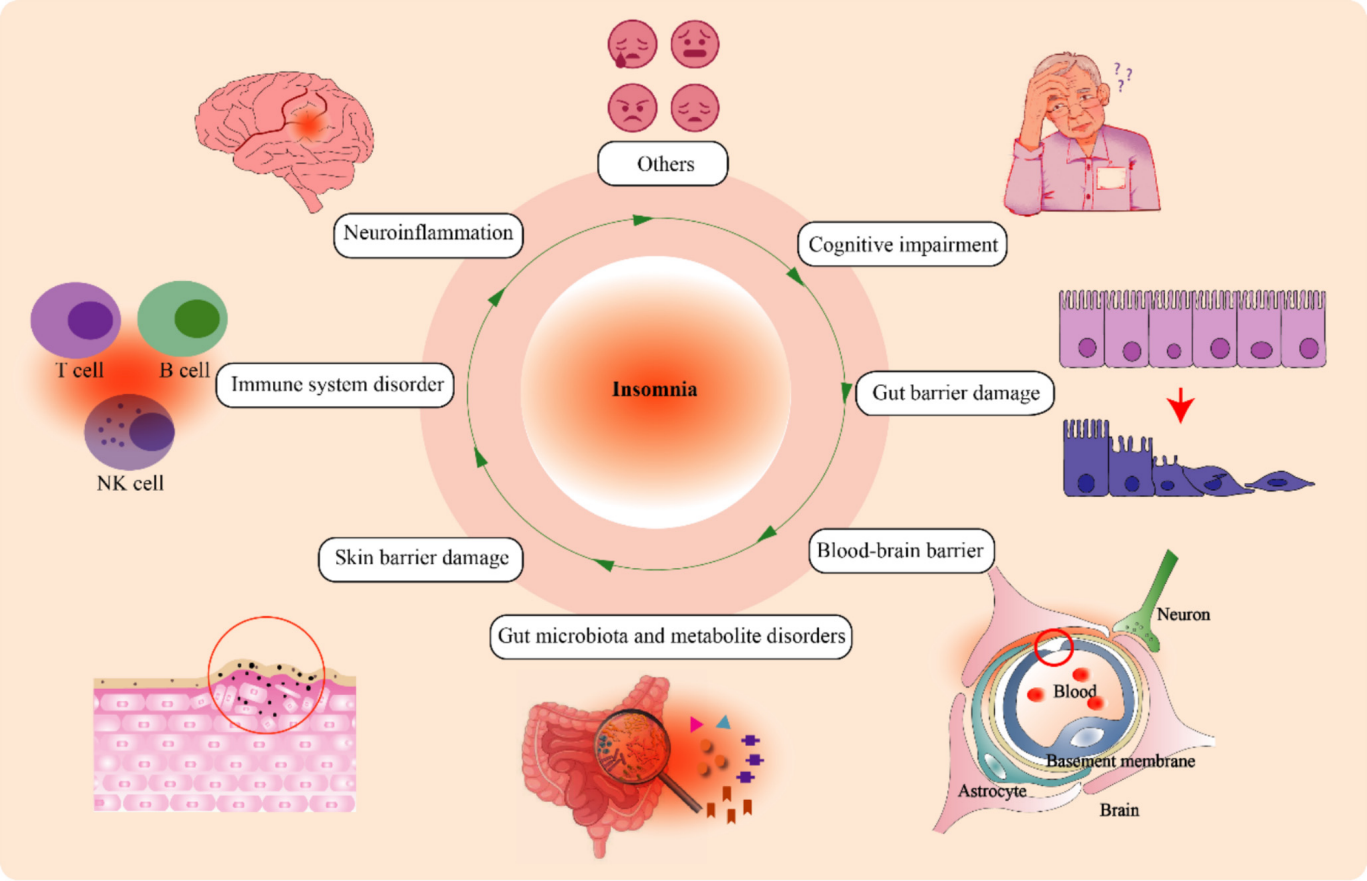
Adverse effects of insomnia on body function. Insomnia leads to a range of detrimental consequences affecting brain function (neuroinflammation), gastrointestinal function (gut microbiota and metabolite disorders), skin barrier function, and immune system. At the same time, insomnia is a major contributor to emotional problems such as anxiety and depression. These adverse effects are interconnected and influence one another.

Recent research has also shed light on the intricate interplay between insomnia and the gut microbiota, gut microbiota-derived metabolites, and immune factors (Fig. 3). This section aims to explore the multifaceted relationship between insomnia and these interconnected components. The following subsections will delve into the alterations in the gut microbiota and their metabolites in individuals with insomnia, as well as the impact of immune factors on the development and progression of insomnia. Elucidating these complex interactions will provide a deeper understanding of the underlying mechanisms of insomnia and its potential treatments.

### Alterations in gut microbiota in insomnia

Chronic SD has been consistently linked to a range of health issues, encompassing increased inflammation, metabolic disorders, as well as neurological and psychiatric conditions. Notably, these adverse outcomes have been found to be associated with alterations in the composition of the gut microbiota (Fig. 3) [136], [137], [138]. The gut microbiota, a complex community of microorganisms residing in the gastrointestinal tract, plays a pivotal role in maintaining gut homeostasis and barrier function. Emerging evidence suggests that insomnia disrupts this delicate balance, causing gut dysbiosis.

Thirteen observational studies, conducted in both human and rodent models, have provided compelling evidence of significant alterations in gut microbiota composition between individuals without insomnia and those with the condition (Table 2). At the phylum level, Firmicutes, which includes genera like *Lactobacillus* and *Streptococcus*, and *Bacteroides* have been consistently identified as differentially abundant bacterial taxa in these studies, indicating their potential involvement in insomnia-related dysbiosis. The abundance of Firmicutes [139,140], Bacteroidetes [139,141] (in two studies), and Tenericutes [142] (in one study) was found to be reduced in individuals with insomnia and model mice. In contrast, the increased abundance of Proteobacteria [143]. Notably, a prospective observational study observed an increasing trend of Firmicutes in women with poor sleep quality after breast cancer surgery [141]. These conflicting results require further validation. Moreover, higher proportions of the gut microbial phyla Verrucomicrobia and Lentisphaerae have been associated with better sleep quality [144].

**Table 2:** Changes in fecal microbiota, metabolites, and blood indicators related to insomnia.

Medical condition or model	Subject	Sample	Alterations	Reference
Gut microbiota				
Insomnia	Human	Feces	*Bacteroides* ↑ Clostridiales ↓	[175]
Insomnia	Human	Feces	*Lactobacillus, Streptococcus, Lactobacillus crispatus*↑	[149]
Insomnia	Human	Feces	*Bacteroides*, *Blautia* and *Pathobionts* ↑	[148]
			*Faecalibacterium* and other SCFA-producing bacteria (*Roseburia, Prevotella, Lachnospira*) ↓	
Insomnia	Human	Feces	*Lachnospiraceae*, *Corynebacterium* and *Blautia* ↑	[139]
			*Bacteroidetes* and *Firmicutes* ↓	
Insomnia	Human	Feces	*Prevotellaceae*↑ *Bacteroidaceae* and *Ruminococcaceae* ↓	[176]
Insomnia	Human	Feces	*Prevotellaceae* (major contributor to gut inflammation) ↑ *Ruminococcaceae*↓	[177]
SD	Human	Feces	Firmicutes/Bacteroidetes ratio ↑ Tenericutes ↓ *Coriobacteriaceae* and *Erysipelotrichaceae*↑	[142]
Anxiety and depression with insomnia	Human	Feces	*Bacteroides* and *Roseburia* ↑ *Prevotella* and *Bifidobacterium* ↓	[152]
Poor sleep quality after breast cancer surgery	Human (female)	Feces	Firmicutes ↑ Bacteroidetes ↓ *Acidaminococcus* ↑	[141]
SD induced by FMT from SD mouse	Mouse	Feces	*Aeromonas*↑ Firmicutes↓	[140]
Chronic SD	Mouse	Feces	*Lactobacillaceae*↓ *Lachnospiraceae* and *Ruminococcaceae* ↑	[145]
SD induced by multi-platform water bath	Mouse	Feces	*Akkermansia, Bacteroides*, and *Faecalibacterium* ↓ *Aeromonas*↑	[7]
4-Chloro-DL-phenylalanine-induced insomnia	Mouse	Feces	Proteobacteria↑ *Romboutsia*↑ *Lactobacillus* and *Clostridium* sensu stricto↓	[143]

Gut microbiota-derived metabolites or their metabolism				
Chronic Insomnia	Human	Feces	Bile acids; positive correlation between muro cholic acid and norcholic acid, while negative correlation with isolithocholic acid	[154]
Insomnia	Human	Feces	Significant differences in glycerophospholipid metabolism; glutathione metabolism; nitrogen metabolism; alanine, aspartate, and glutamate metabolism; aminoacyl-tRNA biosynthesis pathways.	[149]
Insomnia with short sleep duration	Human (older adults)	Feces	Acetate, butyrate, and propionate, and total SCFAs↑	[178]
SD	Human	Feces	SCFAs (acetic acid, butyrate) ↓	[120]
SD induced by FMT from SD subjects	Mouse	Feces	SCFAs (acetic acid, propionic acid, and butyrate) ↓	
SD induced by FMT from SD subjects	Mouse	Feces	Butyrate↓	[140]

Immune factors				
SD	Human	Blood	IL-6 ↑	[139]
SD	Human	Blood	TNF-α, IL-6, IL-1β↑ IL-10↓	[120]
SD induced by FMT from SD subjects	Mouse	Blood	TNF-α, IL-1β and IL-6↑ IL-10↓	
SD induced by multiple platform water bath	Mouse	Blood	IL-1β, IL-6, and TNF-α↑ IL-5, IL-10, and IFN-γ↓	[7]
Insomnia	Human	Blood	IL-1β ↑ TNF-α↓	[149]

Other blood indicators				
SD	Human	Blood	Zonulin and S100β↑ LPS, LPS-binding protein↑ CD14 ↓	[120]
SD induced by FMT from SD subjects	Mouse	Blood	LPS↑ and S100β↑	[120]
Chronic insomnia disorder	Human	Blood	Diamine oxidase, D-lactic acid, Endotoxin, Intestinal fatty acid binding protein ↓	[173]
Depression or anxiety	Human	Blood	Cortisol ↑	[179]
Insomnia	Human	Blood	Brain-derived neurotrophic factor↓	[153]
SD induced by inverted flowerpot technique	Mouse	Blood	Corticosterone↑	[180]
SD	Mouse	Blood	Ghrelin, glucagon-like peptide 1 ↑ leptin ↓	[181]
Night shift workers	Human	Blood	Probiotic supplementation associated with changes in pentraxin, serum cortisol, and mucosal vascular addressin cell adhesion molecule 1	[182]

Note: The symbols '↑' and '↓' represent the increase and decrease of the substance or species level, respectively.

Abbreviations: FMT means fecal microbiota transplantation; IL means interleukin; IFN means interferon; LPS means lipopolysaccharides; SD means sleep deprivation; SCFA means short-chain fatty acid; TNF means tumor necrosis factor.

At the family level, significant differences in abundance were observed among several bacterial families between healthy human or animal subjects and those with insomnia. Specifically, *Lachnospiraceae* [139,145] (in two studies), *Coriobacteriaceae*, and *Erysipelotrichaceae* [142] (in one study) exhibited increased abundance in insomnia subjects or animal models. Conversely, *Lactobacillaceae* [145] (in one study) showed decreased abundance. *Ruminococcaceae,* a prevalent family in the order Clostridiales known for its role in maintaining gut health [146], was found to be significantly reduced in two clinical studies. Therefore, it is reasonable to assume that gut health is impaired in insomnia population. However, an opposite result was observed in one animal model. Notably, conflicting results regarding *Prevotellaceae*, a family associated with gut inflammation, were reported in two insomnia clinical studies, with inconsistent changes observed among insomnia participants. Additionally, in a cohort of elderly individuals with insomnia, the family *Monoglobaceae* and its genus *Monoglobus* were found to be positively associated with sleep efficiency [147].

At the genus level, several significantly differential genera among insomnia patients and healthy individuals were reported, including *Lactobacillus*, *Streptococcus*, *Bacteroides*, *Lachnospira*, *Faecalibacterium*, *Roseburia*, *Prevotella*, *Bifidobacterium*, *Acidaminococcus*, *Aeromonas*, *Akkermansia*, *Elusimicrobium*, *Anaeroplasma*, *Clostridium* sensu stricto, and *Blautia*. Specifically, *Lachnospira* and *Bacteroides* were identified as marker bacteria distinguishing between subjects with acute insomnia from healthy controls, while *Faecalibacterium* and *Blautia* differed between individuals with and without chronic insomnia. Additionally, *Lachnospira*, *Faecalibacterium*, *Bacteroides*, and *Blautia* were associated with plasma IL-1β levels and PSQI scores [148].

Among the genera studied, *Streptococcus* (in one study) [149], *Acidaminococcus* (in one study) [141], *Aeromonas*, and *Blautia* (in two studies) [139,148] exhibited increased abundance in insomnia subjects or animals. Conversely, *Bifidobacterium*, *Lachnospira*, *Elusimicrobium*, *Anaeroplasma* (in one study), and *Prevotella* (in three studies) were observed at lower levels. The increase of *Streptococcus* and the decrease of *Prevotella* and *Lachnospira* were also observed in the fecal microbial community of ulcerative colitis patients with depression/anxiety [150]. *Roseburia*, known for metabolizing dietary components and producing short-chain fatty acids (SCFAs), exhibited conflicting findings, with one clinical study reporting an increase and another reporting a decrease. This suggests that different insomnia conditions may lead to distinct gut microbiota structures [151]. Importantly, the specific microbial taxa associated with insomnia identified in clinical studies often do not overlap, emphasizing the need for large-scale gut microbiota analyses in specific populations, such as perimenopausal women or chronic insomnia patients without other diseases, in future research. Furthermore, conflicting results were found regarding *Lactobacillus* and *Lactobacillus crispatus*, as they were found to increase in one clinical study [149] but decrease in PCPA-induced insomnia animal models [143]. Similar discrepancies were observed for *Bacteroides* [7,152], which may be attributed to inherent differences between human and animal gut microbiota. Furthermore, the potential use of tongue characteristics and oral microbial profiles as diagnostic biomarkers for insomnia has been explored. A higher relative abundance of Clostridia, *Veillonella*, *Bacillus*, and *Lachnospiraceae* were found in the oral microbiota of insomnia patients compared to healthy individuals, and some taxa, including Actinobacteria, Clostridia, and unclassified *Lachnospiraceae ,* were suggested as potential buccal microbial biomarkers for insomnia [153].

These findings underscore the complexity and heterogeneity of the gut microbiota alterations observed in individuals with insomnia, emphasizing the need for further research to elucidate the precise role of specific microbial families and their potential impact on sleep-related outcomes. The gut microbiota plays an important role in maintaining overall health, and gut dysbiosis associated with insomnia can exert adverse effects on the host. Consequently, it represents a valuable diagnostic target for insomnia.

### Alterations in gut microbiota-derived metabolites in insomnia

The disruption of the gut microbiota would naturally impact the gut microbial metabolism and the resultant metabolites, which are key mediators connecting the gut-brain axis and its function. These gut microbiota-derived metabolites encompass various compounds, such as SCFAs, bile acids, amino acids, peptides, peptidoglycans, lipopolysaccharides (LPS), and bacteriocins. Current research findings provide support for the implication of SCFAs, bile acids, and LPS in insomnia (Fig. 3).

Evidence from an independent cross-sectional cohort of 6122 individuals suggests that the gut microbiota-bile acid axis plays a crucial role in establishing a connection between chronic insomnia and cardiometabolic diseases. Specifically, the study found that murocholic acid and norcholic acid exhibit positive correlations with chronic insomnia, while isolithocholic acid shows a negative correlation [154]. These findings implicate that targeting the microbiota-bile acid axis could potentially mitigate the cardiometabolic effects associated with chronic insomnia. Furthermore, a study conducted in SD model mice revealed a strong association between bile acid levels and insomnia. The result showed a significant decrease in colonic chenodeoxycholic acid levels, accompanied by significant increases in tauro-α-murocholic acid and β-muricholic acid levels. Deoxycholic acid and lithocholic acid, two bile acids synthesized in the liver through gut microbiota catalysis, undergo dehydroxylation. Microorganisms involved in this process include *Lactobacillus*, *Bifidobacterium*, *Enterobacter*, *Bacteroides*, and *Clostridium* [155]. These findings provide further support for the notion that insomnia disrupts gut microbiota, impacting bile acid metabolism. However, it is important to note that the causal relationship between microbial metabolites like bile acids and insomnia require further investigation due to the limited number of validation experiments conducted in this field.

Lipopolysaccharide is a crucial component found in the cell wall of Gram-negative bacteria. It is a mediator in microbial-host interactions, found abundantly in and released from the gut and can initiate a series of events that disrupt the inflammatory response, causing both systemic and neuroinflammation, as well as memory impairment [156,157]. Generally, the inflammation process is initiated by the activation of NF-κB transcription through the endotoxin metabolic pathway, involving the binding of LPS to LPS-binding protein and the subsequent transfer to receptor CD14. The LPS-LPS-binding proteinCD14 complex then binds to toll-like receptor 4 (TLR4) and initiates the secretion of proinflammatory cytokines, causing metabolic inflammation [158]. In the context of insomnia, SD has been found to induce gut dysbiosis, trigger inflammatory responses, and result in cognitive impairment in humans (Fig. 3). Interestingly, these effects were suppressed in germ-free mice [120]. And antibiotic treatment further exacerbates cognitive impairment in SD mice [159]. Furthermore, transplanting “SD microbiota” into germ-free mice led to impaired cognitive function, elevated levels of neuroinflammation, and increased microglial activity specifically in the hippocampus and medial prefrontal cortex, two brain regions associated with cognitive processes [120]. These findings highlight the significant role of gut microbiota in mediating the detrimental effects of SD on cognitive function and neuroinflammation. A separate study showed that SD-associated inflammation, cognitive impairment, and intestinal microbiota disturbances can be mitigated by exogenous supplementation of Mel [140]. The authors proposed that Mel decreases LPS levels and promote intestinal beneficial microbes and butyrate levels. These changes alleviate inflammation and neuronal apoptosis in the hippocampus through regulating the TLR4/NF-κB signaling pathway. Such results are supported by another study that observed significant improvement in LPS-induced inflammatory responses in astrocytes by administered Ramelteon, a Mel receptor agonist, through inhibiting the TLR4/IκBα/NF-κB p65 axis. The protective effects were confirmed in an in vivo rodent model, suggesting Mel receptor agonist could protect LPS-induced damage in astrocytes, which also play important roles in sleep regulation [160,161]. Additionally, LPS-binding protein inhibits the biosynthesis of monoamines (such as 5-HT and NA), thereby impacting sleep by acting as an endogenous inhibitor of dopamine-β-hydroxylase and aromatic-L-amino-acid-decarboxylase [162].

Short-chain fatty acids are primarily produced through anaerobic bacterial fermentation in the colon and play a vital role in maintaining gut immune function and modulating gut barrier function [163]. Moreover, SCFAs inhibit the production of pro-inflammatory mediators, such as TNF-α and IL-6, in macrophages stimulated by LPS and cytokines [164,165]. Studies have also demonstrated that SCFAs regulate the maturation of microglia and prevent neuroinflammatory processes [166], [167], [168]. SD has been found to decrease the intestinal levels of propionate, acetate, and butyrate [120]. This decrease is accompanied by a reduction in SCFA-producing bacteria in the gut, such as *Roseburia*, *Prevotella*, and *Lachnospira* [148]. Therefore, the decrease in SCFAs may contribute to the inflammatory response mediated by the increase in LPS observed in individuals with insomnia and animal models. However, a study investigating the relationship between fecal SCFA levels and sleep continuity in elderly individuals with insomnia found that higher concentrations of acetate, butyrate, propionate, and total SCFAs were associated with lower sleep efficiency and longer sleep onset latency [147]. Among that, butyrate is also thought to be a key substance in the regulation of sleep [169]. These conflicting results may be due to variations in subject physiological factors, such as age, gender, body mass index, physical activity, underlying diseases and medications, between studies. Propensity score matching analysis can be utilized to account for confounding factors, allowing for fair comparisons between studies.

Furthermore, multi-omics analysis has revealed five differential metabolic pathways in between individuals with and without insomnia, including glycerophospholipid metabolism, glutathione metabolism, nitrogen metabolism, alanine, aspartic acid, and glutamate metabolism, as well as aminoacyl-tRNA biosynthesis [149]. In summary, current literature has shown significant changes in gut microbial metabolism in individuals with insomnia. The disruption of gut microecology often leads to inflammation and can worsen insomnia symptoms and overall health. Therefore, restoring deficient metabolites like SCFAs and bile acids may potentially alleviate insomnia symptoms and improve the well-being of affected individuals.

### Alterations in immune and other factors in insomnia

Clinical and animal studies consistently demonstrate that SD or insufficient sleep leads to a sustained activation of the inflammatory response. This is characterized by significantly increased blood levels of C-reactive protein and other pro-inflammatory factors, such as IL-1β, IL-6, and TNF-α, accompanied by decreases in anti-inflammatory factors, including IL-5 and IL-10. Additionally, SD is associated with elevated levels of LPS, LPS-binding protein, and cortisol, while soluble CD14 decreases (Table 2). Experimental studies have further shown that both partial and complete SD can activate intracellular signaling pathways involved in inflammation and up-regulate IL-6 and TNF-α transcription [170,171]. Although a meta-analysis reported that sleep disturbances and duration may not be directly associated with TNF-α levels, it is worth noting that TNF-α and IL-1β released by immune cells in the gut can interact with the central nervous system and affect regions responsible for regulating wakefulness, such as the preoptic area of the hypothalamus. Such interaction has potential to modify the firing pattern of neurons in the hypothalamus and brainstem, ultimately influencing brain arousal activity [172]. Furthermore, a negative correlation was found between changes in blood IL-1β levels in insomnia patients and specific microbial species, specifically *Prevotella amnii*, *buccalis*, *timonensis*, and *colorans* [149]. These findings suggest that insomnia-induced inflammatory responses are regulated by the gut microbiota and metabolites.

Additionally, insomnia has been found to disrupt gut barrier integrity and blood–brain barrier (BBB) permeability (Fig. 3), as evidenced by increased levels of zonulin and S100β, and decreased levels of damine oxidase, D-lactic acid, Endotoxin, and intestinal fatty acid binding protein [120,173]. An important study found that metabolic disturbances during SD lead to α-ketoglutarate accumulation in the colon. Excess prolyl hydroxylase 2 degrades hypoxia inducible factor 1 protein, leading to impairment of its intestinal repair function, which may be a potential mechanism for SD damage to the gut barrier [174]. These findings highlight the complex relationship between insomnia, gut barrier integrity, BBB permeability.

While the role of gut microbiota in neuronal regulation is increasingly supported by evidence [159,176], for directly identifying the gut microbiota as an important contributor to insomnia, modelling experiments using germ-free mice or faecal microbiota transplants can provide valuable insights. Overall, the impact of sleep problems on an individual's health is multifaceted and not limited to gut microbiota and metabolites. It disrupts physiological rhythms and weakens functions such as the immune system. Adequate sleep is therefore critical to maintaining overall health and improving quality of life. Future research should continue to investigate the long-term effects of insomnia and develop effective interventions to reduce its impact on public health. By raising public awareness of the importance of sleep and improving sleep hygiene, we can expect to see progress in the prevention and management of health problems associated with insomnia with probiotics.

### Probiotic and postbiotic interventions in insomnia

The above results reveal a clear interactive relationship between gut microbiota and insomnia. At the same times, there is a direct link between the composition of the gut microbiota and the probiotics. Many probiotics were originally isolated from the gastrointestinal tract and are defined by the FAO/WHO as “living microorganisms that, when given in sufficient amounts, are beneficial to the health of the host” [183]. In addition, postbiotic are defined as a “preparation of inanimate microorganisms and/or their components that confers a health benefit on the host” [184] and likewise have a positive impact on the gut microbiota [185]. Thus, an emerging trend is to explore whether probiotics and postbiotics have favorable biological activities to improve insomnia.

### Rodent intervention trials

Animal models provide an effective approach to investigating the role of probiotics, with insomnia. Insomnia animal models are constructed in two broad ways: stress intervention and chemical intervention (Table 3). Stress interventions induce insomnia by using methods like, roller, multiple-platform water bath, and electric shock. Chemical intervention in insomnia animal models primarily involves the use of substances, such as PCPA, caffeine. PCPA intraperitoneal injection to induce insomnia is currently the main modality of modelling.

**Table 3:** Probiotic effects in animal intervention trials.

Animals	Insomnia induction method	Probiotic(s) or other product, dose, and intervention duration	Major effects	Sleep assessment method(s)	Reference
Male C57BL/6J mice (6 weeks old)	Caffeine (15 mg/kg)	*Limosilactobacillus fermentum* PS150TM, 8 × 10^10^ CFU/kg/day, 14 days	Strain-specific sleep improvement effect, reduction in caffeine-induced sleep disorders in mice	Pentobarbital-induced sleep test	[190]
Male sprague-dawley rats (180–220 g)	4-Chloro-DL-phenylalanine, 400 mg/kg, 2 days	*Armillaria mellea* fermentation liquor, 7 days	Improved insomnia by regulating serotoninergic system and gut microbiota	Pentobarbital-induced sleep test	[188]
Male institute of cancer research mice (6 weeks old, 25–30 g)	−	Probiotic fermented milk produced by *Levilactobacillus brevis* and *Lactiplantibacillus plantarum*, 30 days	High-dose of GABA in the fermented milk improved sleep, through regulating gut microbiota and increasing short-chain fatty acids	Pentobarbital-induced sleep test	[191]
Male C3H/HeN mice (5 weeks old)	Wheel-running activity	Heat-killed *Levilactobacillus brevis* SBC8803, 42 days	Improved stress-induced insomnia, circadian rhythm sleep disorders, and non-rapid eye movement sleep	Electroencephalogram (EGG)	[195]
Male C57BL/6J mice (6 weeks old)	Cage change	*Limosilactobacillus fermentum* PS150, 9.75 × 10^8^ CFU/kg/day, 28 days	Reduced sleep onset latency, restored normal rapid eye movement sleep, and improved sleep disorders caused by familial nocturnal epilepsy; accompanied by increases in fecal Erysipelotrichia, Actinobacteria, and Coriobacteriia	Polysomnography	[196]
Male B6128SF2 mice (8 weeks old, 25–35 g)	Automated sleep deprivation chamber, 7 days	Multi-strain probiotic formulation, 2 × 10^11^ CFU/kg/day, 60 days	Improved the antioxidant capacity of the brain, thereby limiting SD-induced oxidative damage. Sleep quality not mentioned.	Direct visual observation	[181]

Note: We standardised the dose of probiotic administration to 0.025 kg of body weight for mice and 0.25 kg for rats.

Intraperitoneal injection of PCPA has been widely utilized as a selective and irreversible inhibitor of tryptophan hydroxylase that effectively inhibits 5-HT synthesis and has a high success rate in establishing insomnia models [186]. This method enables the assessment of whether interventions can alleviate insomnia resulting from disruptions in the 5-HTergic system. In addition, insomnia-induced by PCPA is commonly employed in animal studies (Table 3). However, it is important to note that PCPA-induced insomnia is a reversible condition, and generally, insomnia symptoms persist for only a relatively short period of nine to 14 days following PCPA administration [187]. It is worth attempting to prolong the insomnia effect by varying the PCPA administration time and dosage. Nevertheless, the efficacy of only 7 days of *Armillaria mellea* fermentation liquor consumption in improving sleep quality through modulation of the 5-HT system and gut microbiota was demonstrated in rats with PCPA-induced insomnia in a previous intervention study [188]. Of course, more research is needed to see if short-term probiotic interventions improve sleep.

Rodent studies have also played a crucial role in uncovering the molecular mechanisms that regulate the sleep cycle. For example, a previous work analyzed differentially expressed genes during the sleep cycle, leading to the identification of an astrocyte brain-type fatty acid binding protein, FABP7, which plays a crucial role in lipid metabolism and is required for maintaining normal sleep. Knocking out the FABP7 gene in mice results in intermittent sleep patterns [189]. Knocking out these genes may provide a potential means to constructing insomnia rodent models for assessing the beneficial effects of probiotics or other related products on sleep quality.

Sleep quality assessment in rodents is primarily done using invasive electroencephalogram/electromyogram techniques and pentobarbital-induced sleep test (Table 3) [188,190,191]. However, these methods have drawbacks like sleep disruption, postoperative recovery time, procedure-related infections or fatalities, and issues like electrode misalignment or noisy signals. Noninvasive sleep monitoring systems like PiezoSleep, which uses a sensing pad beneath the cage floor to monitor mouse movement, have gained interest due to their potential. The system uses SleepStats software to analyze vibration changes detected by the sensing pad and wakeful breathing patterns [192,193]. The system has been validated using implanted electrodes in mice and rats [192], offering a reliable alternative for automatic and visual sleep-wake scoring. However, it has not been used in studies assessing insomnia improvement related to probiotic intervention. Pentobarbital-induced sleep test is one of the methods for evaluating the effectiveness of sleep-enhancing substances, as outlined in the Technical Code for the Inspection and Evaluation of Health Foods (2003 edition). This method assesses whether the test substance can extend the duration of sleep induced by pentobarbital. Prolonged sleep duration indicates a sleep-promoting effect of the test substance. One previous study found that fermented milk produced by a high-yield GABA probiotic strain could increase the sleep duration induced by pentobarbital sodium, accompanied by reduced sleep onset latency, indicating the sleep-prolonging effect of this bacterial strain [191]. Moreover, the open field test can also be used as an indirect method for evaluating insomnia [194].

Animal studies on assessing probiotic potential to improve insomnia are limited due to challenges such as the limited duration of drug modeling, spontaneous recovery, and the subjective nature of sleep quality assessment. The PiezoSleep system holds promise for future sleep research, but more reliable methods for assessing sleep quality are needed to fully understand the mechanisms involved in using probiotics to enhance insomnia.

## Human intervention trials

In addition to studies conducted in rodents, a large number of studies are focusing on insomnia populations, exploring the anti-insomnia, sleep-quality improvement, and anti-stress effects of probiotics in human intervention trials (Table 4). Previous studies have demonstrated that administration of probiotics for three to 12 weeks can significantly improve sleep quality, as evaluated by the PSQI, insomnia severity index (ISI), and Athens insomnia scale, with concurrent improvements in anxiety, stress, depression, and cognitive function [109,152,197]. A double-blind, placebo-controlled, randomized study found that consuming *Lactobacillus acidophilus* Rosell-52 or *Bifidobacterium longum* Rosell-175 did not improve any physiological or psychological symptoms or sleep problems related to stressful events [198]. These findings suggest that not all probiotics have a beneficial effect on insomnia, and there might be functional specificity among different probiotics. It is important to note that the aforementioned studies were conducted solely relying on subjective scales to assess sleep, lacking objective accuracy.

**Table 4:** Human intervention studies of probiotic effects on subjects with insomnia or other-related conditions.

Subjects (sample size)	Probiotic strain(s) or product	Dose, intervention duration			Major effects	Subjective assessment		Objective assessment	Reference
Subjects with insomnia or sleep problems									
Men with mild insomnia (n = 40)		Heat-killed *Levilactobacillus brevis* SBC8803	10 days	Sleep diary reveal that slight improvement in wakefulness and drowsiness scores on the second day after SBC8803 intake. No statistical difference in EEG sleep analysis or AIS.		Sleep diary, AIS		EGG	[209]
Healthy subjects with sleep problems (n = 21)		Foods containing lactic acid bacteria components extracted from *Lactobacillus helveticus* MIKI-020 and theanine	28 days	Improvements in objective sleep evaluation parameters, including physical fatigue, enthusiasm (vitality), and sedation levels. No improvement in PSQI and Oguri Shirakawa Azumi.		PSQI, Oguri Shirakawa Azumi		EGG	[213]

Subjects with anxiety, depression, or high stress level									
Depression, anxiety and insomnia (n = 156)	*Limosilactobacillus reuteri* NK33 and *Bifidobacterium adolescentis* NK98	8.3 × 10^7^ CFU/kg/day, 56 days			Significantly reduced depression and anxiety levels. Improved sleep quality (PSQI). Decreases in IL-6 level and the abundance of *Enterobacteriaceae*. Increased abundances of *Bifidobacterium* and *Lactobacillus.*	PSQI, AIS and ISI		−	[109]
Anxious students (n = 60) and non-anxious students (n = 30)	*Lactiplantibacillus plantarum* JYLP-326	2.5 × 10^8^ CFU/kg/day, 21 days			Significantly improved anxiety and depression levels. Insomnia was positively correlated with anxiety and depression levels.	AIS		−	[152]
High pressure information technology experts (n = 36)	*Lactiplantibacillus plantarum* PS128TM	3.33 × 10^8^ CFU/kg/day, 56 days			Significantly improved self-perceived stress, cortisol levels, anxiety, depression, sleep disorders, quality of life, and negative emotions.	ISI		−	[214]
Stressful students (n = 30)	*Levilactobacillus brevis* PBS072 and *Bifidobacterium breve* BB077	6.67 × 10^7^ CFU/kg/day, 28 days			Improved stress resilience, cognitive function, and sleep quality.	AIS		−	[197]
High pressure medical students (n = 24)	*Lactobacillus gasseri* CP2305	1.67 × 10^8^ CFU/kg/day, 28 days			Significantly improved sleep quality.	PSQI		−	[215]
Healthy students (21 males and 11 females) in an autopsy course	Heat-killed *Lactobacillus gasseri* CP2305 milk beverage	35 days			Shortened sleep onset latency and increased total sleep time.	PSQI		−	[216]
Healthy participants (n = 47)	*Lactocaseibacillus casei* Shirota YIT9029	1.67 × 10^9^ CFU/kg/day, 56 days			No difference in PSQI score between placebo and intervention groups.	PSQI		−	[217]
Students facing test pressure (n = 94)	*Lactocaseibacillus casei* Shirota fermented milk	1.67 × 10^8^ CFU/kg/day, 77 days			Prevented the decrease in non-rapid eye movement sleep in the third stage and the increases in δ power by more than 20 %, drowsiness, and total sleep time (EGG, OSA).	Oguri Shirakawa Azumi, PSQI		EGG	[199]
Stress symptoms (n = 75)	*Lactobacillus acidophilus* Rosell-52 and *Bifidobacterium longum* Rosell-17	5 × 10^7^ CFU/kg/day, 21 days			Did not significantly improve sleep problems and other stress-related physiological and psychological symptoms.	Questionnaire		−	[198]
Healthy participants (n = 60)	*Lactobacillus gasseri* CP2305 (n = 29) or a placebo (n = 31)	1.67 × 10^8^ CFU/kg/day 24 days			Shortened sleep onset latency and wake-up time after sleep. Increased δ power ratio in the first sleep cycle (EGG, PSQI). Alleviated stress-induced decrease in *Bifidobacterium* and increase in *Streptococcus*.	PSQI		EEG	[218]
Night shift workers (n = 87)	*Lactobacillus acidophilus* DDS-1 and *Bifidobacterium animalis* subsp. *lactis* UABla-12	1.67 × 10^8^ CFU/kg/day 14 days			Significantly improved PSQI, but no difference in total sleep time.	PSQI		Fitbit activity tracker	[182]

Subjects with other conditions indirectly related to insomnia									
Patients with irritable bowel syndrome (n = 42)	Probiotics VSL # 3 (*Bifidobacterium longum*, *Bifidobacterium infantis*, *Bifidobacterium breve*, *Lactobacillus acidophilus*, *Lacticaseibacillus casei*, *Lactobacillus delbrueckii*, *Lactobacillus bulgaricus* and *Lactiplantibacillus plantarum,* and *Streptococcus salivarius* subsp. *thermophilus*	1.875 × 10^9^ CFU/day, 42 days			Improved irritable bowel syndrome-associated symptoms and increased salivary melatonin level. Improved sleep scores (PSQI, ESS).		PSQI, ESS	−	[219]
Infants with colic (n = 80)	*Bifidobacterium animalis* subsp. *lactis* BB-12® and DSM 15954	5 × 10^7^ CFU/kg/day, 28 days			Increased the abundance of *Bifidobacterium* and total sleep time.	Sleep diary		−	[220]
Subjects with surgical removal of tooth (n = 61)	*Limosilactobacillus reuteri* (DSM 17,938 and ATCC PTA 5289)	3.34 × 10^6^ CFU/kg/day, 14 days			Reduced frequencies of sleep disorders and interruptions.	Self-reported data		−	[211]
Myalgia encephalomyelitis/chronic fatigue syndrome (n = 40)	*Lacticaseibacillus rhamnosus* *Bifidobacterium lactis* *Bifidobacterium breve* *Bifidobacterium longum*	2.45 × 10^5^ CFU/kg/day, 28 days			No difference in objective sleep evaluation measurement, but improved subjective sleep evaluation parameters and sleep quality. Reduced wake up time at night.	Sleep diary		Wrist Actiwatch monitors	[221]
Colic cohort (n = 99) and non-colic cohort (n = 182)	*Limosilactobacillus reuteri* DSM 17938	−			No effect on the sleep quality.	Brief Infant Sleep Questionnaire		−	[222]

Healthy subjects									
Healthy participants (n = 33)	Probiotic mix (*Limosilactobacillus fermentum* LF16, *Lacticaseibacillus rhamnosus* LR06, *Lactiplantibacillus plantarum* LP01, and *Bifidobacterium longum* BL04)	6 × 10^7^ CFU/kg/day, 42 days			Reduced depression, anger, and fatigue. Improved sleep quality.	PSQI		−	[210]
Healthy men (n = 20)	*Bifidobacterium longum* AH1714	1.67 × 10^7^ CFU/kg/day, 56 days			No difference in PSQI results. Improved total sleep time.	PSQI		−	[211]
Healthy elderly subjects (n = 29)	*Lactobacillus helveticus* CM4 fermented milk	21 days			Significantly improved subjective evaluation parameters and waking times (Actigraphy), but no significant difference in sleep health risk index.	Sleep-Health Risk Index		Actigraphy	[201]
Healthy subjects (n = 29)	*Lacticaseibacillus rhamnosus* (JB-1)	10^9^ CFU/day, 56 days			No difference in PSQI and EGG results. No overall effects on mood, anxiety, stress, and sleep quality.	PSQI		EGG	[208]
Healthy subjects (good sleep quality) (n = 68)	Japanese sake yeast supplementation	4 days			Increased δ power in the first cycle of slow wave sleep, but no difference in other sleep parameters.	EGG		−	[212]

Note: We standardised the dose of probiotics to be taken at 60 kg for adults and 20 kg for children.

Abbreviations: AIS means athens insomnia scale; EGG means electroencephalogram; insomnia severity index means ISI, PSQI means pittsburgh sleep quality index, ESS means Epworth sleepiness scale.

Several studies utilize sleep monitoring equipment to provide a more objective evaluation of sleep quality among subjects. Some of them focused on evaluating the benefits of probiotic or postbiotic consumption. For example, a study using electroencephalogram and Oguri Shirakawa Azumi sleep inventory score to assess sleep found that *Lacticaseibacillus casei* Shirota intake prevented a decrease in NREM sleep and increased δ power by over 20 %, suggesting consuming this probiotic strain could improve stress-induced insomnia [199]. Another study showed that ingesting inactivated *Lactobacillus gasseri* CP2305 for 12 weeks significantly enhanced sleep quality, as evaluated by electroencephalogram and PSQI [200]. Similarly, sleep improvement effects in prolonged sleep efficiency and decreased waking times have been observed following the intervention with *Lactobacillus helveticus* CM4 fermented milk, monitored by actigraphy, However, subjective monitoring data did not demonstrate the same level of improvement [201]. Overall, the use of electroencephalogram offers a more objective evaluation of sleep quality, particularly by providing objective information such as sleep efficiency, number of awakenings, and sleep structure. Objective indicators obtained through electroencephalogram can offer a detailed understanding of changes in sleep structure, especially when subjective scales do not show significant improvements. However, Absolute and relative sleep time, absolute and relative wake time after sleep onset, sleep onset latency and sleep efficiency based on actigraphy recordings were not affected by the probiotic intervention [202]. The results of a meta-analysis based on 14 studies found that probiotics or paraprobiotics significantly reduced PSQI scores (−0.78 points, 95 % confidence interval: 0.395–1.166; *P* < 0.001), but otherwise no significant effects were found for changes in other subjective sleep scales, nor objective parameters of sleep measured using polysomnography or actigraphy [203]. Not only that, the effect of microbiota modulation showed no significant improvement on sleep quality in another meta-analysis (*P* = 0.31) [204]. Therefore, to improve sleep with probiotics or postbiotics, further refinement of evaluation and analysis methods is needed.

Among the different probiotic strains tested, *Lactobacillus gasseri* CP2305 has emerged as a primary focus in numerous insomnia-related studies. This particular strain has been shown to restore gene expression in important protein pathways, such as EIF2 and mTOR signaling pathways [205]. Moreover, Additionally, it has been observed to contribute to the balance between sympathetic and parasympathetic activity, which is crucial for sleep regulation [200]. A meta-analysis found that CP2305 intake significantly improved sleep quality in seven studies, with at least half of the results showing enhancement after consumption based on the assessment on the PSQI scores and/or electroencephalogram [206]. These findings suggest that probiotics, now subclassed as psychobiotics, play a role in regulating sleep patterns in individuals with normal circadian rhythms.

Some postbiotic intervention studies also showed promising effect of postbiotics on insomnia improvement (Table 4). For example, a combination of *Lactobacillus helveticus* MIKI-020 postbiotic and theanine, known for its relaxing effects, has shown improvements in sleep efficiency. Similarly, inactivated *Lactobacillus gasseri* CP2305 has demonstrated a sleep-enhancing effect [207]. However, other studies found that administering inactivated *Lacticaseibacillus rhamnosus* JB-1 [208] or *Levilactobacillus brevis* SBC8803 [209] did not show significant improvements in sleep quality although some of these strains showed ameliorative effects on insomnia and circadian rhythm sleep disorders in mice. The discrepant results suggest that probiotic and postbiotic effects are host- or species-specific. The variations in beneficial effects between studies may implicate the effects of bacterial strains, form of the product (administered as probiotic or postbiotic; pill, dried powder or probiotic fermented milk), dose, intervention duration, and inter-species differences.

Other factors, including the underlying cause of insomnia and its potential association with mood or stress-related issues, may influence the effectiveness of probiotic or postbiotic products in improving symptoms. Currently, there is limited research on insomnia in healthy individuals, with only five studies addressing this specific population [201,208,[210], [211], [212]. In these studies, “healthy subjects” generally refers to individuals without significant sleep-affecting diseases or mental disorders. However, the findings from these two studies exhibit inconsistencies when subjective and objective indicators were used to assess sleep quality (Table 4). These inconsistent results suggest that the choice of sleep evaluation methodologies may impact the results of determining the efficacy of probiotic or postbiotic interventions in improving sleep quality.

It is worth noting that most current research on probiotic or postbiotic intervention have primarily focused on evaluating treatment efficacy rather than analyzing the underlying molecular mechanisms of sleep improvement. Understanding the beneficial mechanisms on the molecular level is crucial for identifying potential probiotic candidates in the future and designing of non-conventional drug based-therapies for managing insomnia. This mutual interaction between the gut microbial community and insomnia also offers new avenues for developing insomnia management strategies based on probiotics or postbiotics that can modulate host gut microbiota.

## Potential mechanisms of probiotics and postbiotics in sleep regulation

### Reshaping the gut microbiota and metabolome

The human intestine is a remarkably complex neural network, is composed of complex networks of neurons and glia, which are present throughout the gastrointestinal tract. In addition to its primary role in nutrient absorption, the gut plays a significant role in communicating with the brain, influencing mood and behavior through various signaling pathways. Furthermore, some intestinal microbial taxa, such as Clostridiales, Lactobacillales, Bacteroidales, Bacteroidetes, and Firmicutes, exhibit circadian fluctuations [223], [224], [225]. Likewise, gut microbial metabolites like SCFAs and butyrate also fluctuate rhythmically throughout the day [226,227]. As a result, the gut microbiota, which consists of trillions of bacteria residing in the intestines, is believed to have a broad impact on metabolic, gastrointestinal, and neurological conditions [228], [229], [230], [231].

In 2019, a novel drug called 'GV-971’ [232], also known as sodium mannan, was developed for the treatment of alzheimer's disease. This drug operates by stimulate the gut microbiota to produce essential amino acids and SCFAs [233]. These metabolites then influence the activity of neurotransmitters in the human brain, which is importing for clearing brain waste and maintaining the connections between neurons. The introduction of this medication represents a significant milestone, demonstrating that gut-targeted approaches can now be utilized in the treatment of brain-related disorders. Expanding from this concept, insomnia can lead to alterations in the composition of the gut microbiota and its metabolites, which are linked to sleep disturbances. The intricate relationship between the gut and brain suggests that targeting the gut microbiota through probiotic interventions could potentially alleviate insomnia-related symptoms. Consequently, probiotics hold promise as a promising microecological probiotic agents for modulating sleep by positively influencing the gut microbiota and its metabolites, thereby improving sleep patterns.

Previous intervention studies have provided evidence supporting the beneficial effects of specific probiotic strains in alleviating insomnia symptoms and promoting sleep quality, and the beneficial effects are accompanied by gut microbiota modulation. For example, *Lactiplantibacillus plantarum* JYLP-326 alleviated insomnia symptoms in students with test anxiety, partially reversing gut dysbiosis [152]. In addition to *Lactiplantibacillus plantarum* JYLP-326, other probiotic strains have also demonstrated positive effects on sleep. *Lactobacillus gasseri* CP2305 [215] and the probiotic product, NVP-1704 (comprising two strains, *Limosilactobacillus reuteri* NK33 and *Bifidobacterium adolescentis* NK98) [109] have both inhibited the growth of *Enterobacteriaceae* and improved sleep quality. Notably, NVP-1704 supplementation increased the abundance of intestinal *Lactobacillus* and *Bifidobacterium*. A separate study supplemented *Bifidobacterium animalis* subsp. *lactis* BB-12VR (BB-12), resulting in increased levels of *Bifidobacterium*, significantly associated with reduced infant crying time and increased total sleep time [220]. These findings demonstrate the positive impact of a sufficient level of gut bifidobacteria on improving sleep quality, as well as the potential of specific probiotic strains in restoring a healthier gut microbiota favoring insomnia alleviation. Further research is warranted to explore the mechanisms underlying these effects, particularly the associated changes in functional gut microbial metabolites subsequent to probiotic intake.

**Fig. 4:**
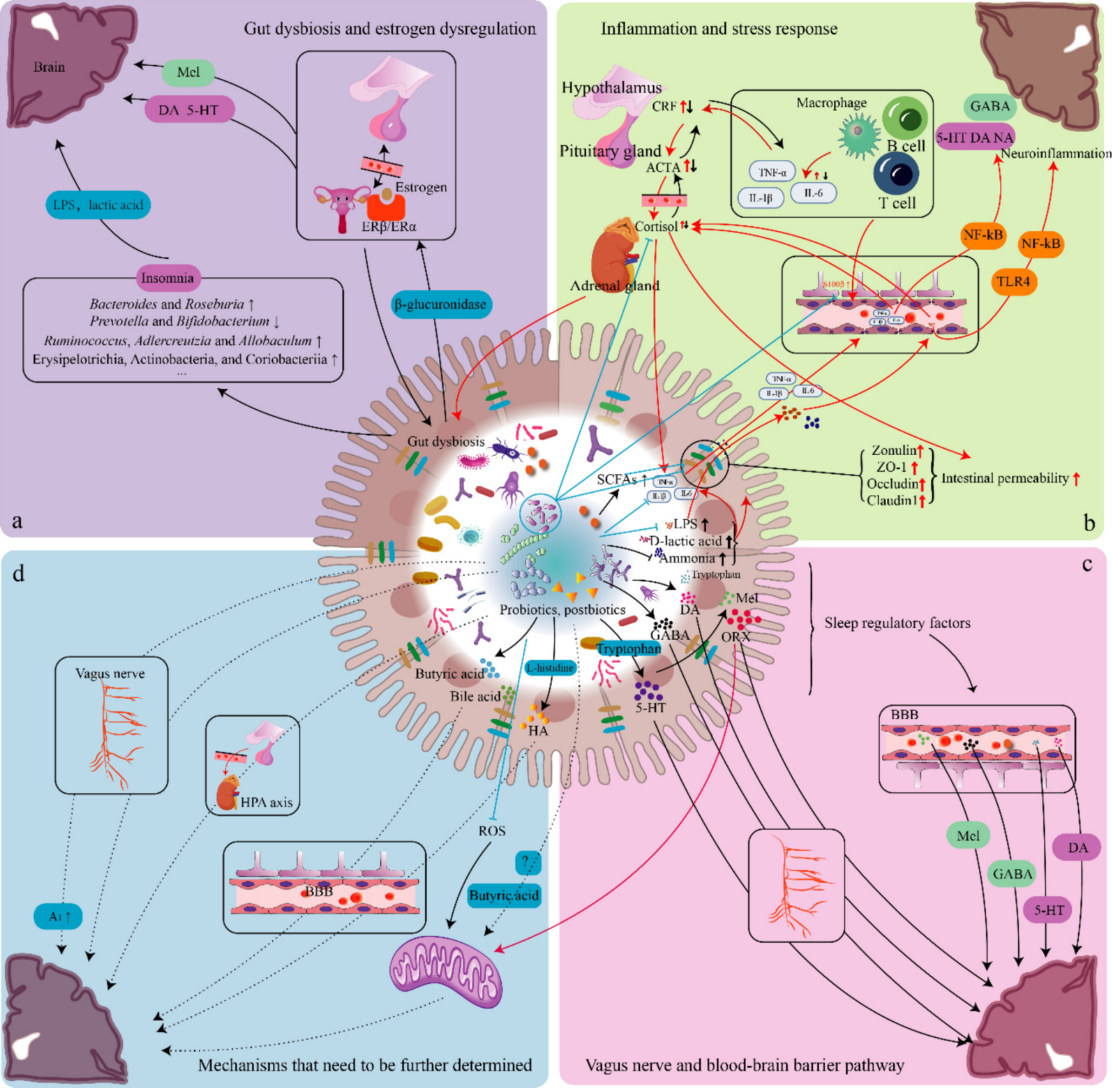
Potential mechanisms of probiotics in sleep regulation. (a) The intake of probiotics can modulate the composition, metabolites of the gut microbiota and the integrity of the gut barrier. Gut dysbiosis can compromise the integrity of the gut barrier, leading to the translocation of LPS and leaking of lactic acid to the systemic circulation and stimulation of production of pro-inflammatory factors. Ultimately, such disruption of the gut barrier function can impact the function of the BBB, allowing harmful gut metabolites to enter the brain, thereby influencing normal brain function. β-glucuronidase being an enzyme responsible for deconjugating estrogen and the decrease in gut microbial diversity can negatively affect β-glucuronidase activity, leading to reduced estrogen levels. Estrogen disorders affect levels of substances such as Mel and 5-HT, thereby affecting the sleep-wake system. (b) Stress response and inflammation can cause damage to the gut barrier as well as disruption of the HPA axis, and even certain lactobacilli can affect cortisol levels. In addition, certain cytokines are regulated by the gut microbiota and are disrupted, ultimately causing neuroinflammation and disruption of certain substances that affect brain function. (c) SRFs such as GABA and 5-HT produced by gut microbiota and enterocytes affect sleep through the BBB as well as vagus nerve pathways. Peripheral orexin in the intestinal mucosa can modulate brain functions. Mel can be synthesized from tryptophan in the gut. (d) There are additional mechanisms that warrant further exploration regarding how probiotics improve insomnia. These mechanisms may involve the effects of probiotics on other metabolites, receptors, and mitochondria. Mitochondria-associated pathways play an important role in sleep, and probiotic interventions can regulate mitochondrial function by inhibiting ROS levels [277]. Butyrate is an important mediator in the regulation of mitochondrial function [278] and orexins are regulators of mitochondrial function and dynamics [279], all of which are associated with the sleep-wake system. In addition, it also remains to be explored whether certain bacterial community, as well as metabolites, influence sleep via the vagus nerve, BBB, or by affecting HPA axis activity. Solid lines indicate pathways that have been identified and dashed lines indicate pathways that are currently unidentified or need to be continued to be explored; red lines indicate facilitation and blue lines indicate inhibition. Abbreviations:5-HT, 5-hydroxytryptamine; A_1_, adenosine receptor; ACTH, adrenocorticotropic; hormone; BBB, blood–brain barrier; CRF, corticotropin-releasing factor; DA, dopamine; IL, interleukin; GABA, γ-aminobutyric acid; LPS, lipopolysaccharides; Mel, melatonin; NA, noradrenaline; NF-kB, tyrosine Kinase receptor B; ORX, orexin; ROS, reactive oxygen species; SCFAs, short-chain fatty acid; SRFs, sleep regulator factors; TLR4, toll-like receptor 4; TNF, tumor necrosis factor; ZO-1, zonula occludens-1.

A previous study showed that supplementing BB-12 resulted in an elevated fecal level of propionic acid in infants with colic, accompanied by extended periods of uninterrupted sleep [234]. Probiotic administration can decrease LPS levels by increasing gut bifidobacteria, and decrease the rate of LPS translocation through the intestinal barrier (Fig. 4) [235]. In a study on mice with alcoholic liver injury, *Lactiplantibacillus plantarum* J26 reduced gut LPS content and decreased the abundance of potential Gram-negative pathogens [236]. As previously described, toll-like receptor 4 (TLR4) plays an important role in mediating LPS-induced microglia activation and inflammatory responses [237]. These findings further implicate probiotic administration can reduce the expression of TLR4 and inhibit the translocation of NF-kB in the brain [238], thereby suppressing the activation of the TLR4-mediated MAPK signaling pathway [239], which in turn reducing neuroinflammation.

Numerous studies have provided evidence supporting the ability of specific gut microbes and probiotics, such as *Lactobacillus*, *Bifidobacterium*, *Escherichia*, and *Enterococcus*, to synthesize neurotransmitters and various SRF, including tryptophan, 5-HT, BDNF, and GABA [24,25,240,241]. In a previous mouse study, insomnia was found to improve only when consuming fermented milk containing 33.33 mg/kg GABA prepared by a high GABA-producing strain of *Lactobacillus,* and was accompanied by significant changes in the gut microbiota and metabolite composition of the mice, including increases in intestinal *Adlercreutzia*, *Allobaculum*, and butyrate [191]. Demonstrate that high enough concentration of functional metabolites would be required to trigger the beneficial effects. In addition, the use of antibiotics that disrupt the gut microbiota has been shown to deplete 5-HT in the gut and affect sleep patterns [242], indicating that 5-HT may play a role in the communication between the gut microbiota and the brain in sleep regulation. Probiotics such as *Levilactobacillus brevis* PBS072 and *Bifidobacterium breve* BB077, which have a higher capacity for synthesizing 5-HT, have demonstrated significant improvements in psychophysiological indices, including salivary cortisol levels, sleep quality, and anxiety [197]. Similarly, another probiotic strain, *Lacticaseibacillus casei* 327, has been found to indirectly increase the production of 5-HT in the colon [26], and the intake of heat-killed *Lacticaseibacillus casei* 327 could improve defecation in healthy adults who have relatively low defecation frequencies [243]. However, whether this strain has any anti-insomnia effect remains to be further investigated.

These findings highlight the role of specific probiotic strains in regulating the gut-brain axis, which is a complex communication network between the gut, microbiota, and the brain. Probiotic supplementation has the potential to restore the disrupted balance of gut microorganisms, microbiota-derived metabolites (such as LPS and SCFA), and other important metabolites like GABA and 5-HT that are affected by insomnia. By modulating these factors, probiotics and their metabolites, known as postbiotics, can directly or indirectly have a positive impact on sleep quality (Fig. 4).

### Enhancing the integrity of gut barrier and BBB

The gut barrier is essential for maintaining gut immunity and protecting against potential pathogens from the external environment [244]. Insufficient sleep can disrupt the integrity of the gut barrier, increasing permeability and causing elevated levels of zonulin [120,245] and decreased expression of tight junction proteins, such as ZO-1, occludin, and claudin-1 [7]. Damage to the gut barrier is linked to gut dysbiosis, stress responses, inflammation-induced reduction of intercellular tight junction proteins, and damage to gut cells. Imbalances in the gut microbiota, caused by toxins or other metabolites like D-lactic acid [221], intestinal ammonia generated from amino acid metabolism [246], antibiotics, and LPS, can trigger the release of inflammatory factors by gut immune cells, compromising the integrity of the gut barrier. Meanwhile, overgrowth of opportunistic pathogens also contributes to gut dysbiosis and disruption of gut tight junctions [246,247]. Excessive cortisol levels can increase gut permeability. Consuming exogenous probiotics and postbiotics can restore the balance of gut microbiota and metabolites, thereby protecting the integrity of the gut barrier. For instance, *Pediococcus pentococcus* PP04 showed enhancement effects on the expression of ileal tight junction proteins, including occludin, claudin-1, and ZO-1 [248]. *Lactiplantibacillus plantarum* J26 has been shown to reduce gut LPS levels, while SCFAs produced by anaerobic bacteria help maintain gut immune function and regulate gut barrier function [165] (Fig. 4b).

The gut, being the body's largest endocrine organ, plays a significant role in releasing various biological signals, such as neurotransmitters or hormones. These signals can enter the bloodstream and directly affect the brain by passing through the BBB, which acts as a selective filter that prevents harmful substances. Insomniacs have elevated serum levels of S100β, and fecal microbiota transplantation from individuals with insomnia to mice increased S100β levels in the recipient mice [120], implicating that the gut microbiota may influence insomnia through BBB, particularly in occasions when the integrity of the BBB is weakened. Neurotransmitters and related signaling molecules can act locally on the enteric nervous system or directly affect the brain through the BBB or vagus nerve. They have the ability to influence brain function and transmit information to nerve cells in the gut. In addition, the gut microbiota releases tryptophan and glutamate into the bloodstream, which can interact with downstream transporters or receptors, thereby promoting the synthesis of 5-HT and GABA, impacting sleep quality [24,25,249]. Senescence-accelerated mouse prone-8 (SAMP8) mice, a naturally occurring mouse strain, display accelerated aging traits that are associated with changes in gene expression and protein abnormalities observed in Alzheimer's disease, including sleep disruptions and reductions in both NREM and REM sleep durations. Probiotics have been shown to significantly reduce BBB damage in SAMP8 mice by restoring balance to the gut microbiota and its metabolites, alleviating inflammation, stress response, and replacing antibiotics [238] (Fig. 4c). In short, vailable experiment evidence suggests that probiotics may improve insomnia by protecting the gut barrier and maintaining BBB integrity.

## Regulating endocrine pathways through the HPA axis

As mentioned above, the HPA axis serves as a vital endocrine pathway that plays a crucial role in the regulation of sleep. Both cortisol and epinephrine exert an influence on the immune cell function and differentiation, as well as on gut microbiota, by modulating cytokine secretion within the HPA axis.

In a study conducted on rats, a diet containing *Lacticaseibacillus casei* Shirota was administered for two weeks. Following exposure to water avoidance stress, the rats' plasma corticosterone levels, as well as the expression of c-Fos (a marker of neuronal activity) and corticotropin-releasing factor in the paraventricular nucleus. The results indicate that *Lacticaseibacillus casei* Shirota administration could modulate the activity of the HPA axis and suppress corticosterone secretion under stressful conditions [250]. Another study involving the use of heat-inactivated *Lactobacillus gasseri* CP2305 revealed its ability to improve sleep while preventing the release of salivary cortisol and the expression of stress-responsive microRNAs (miR-144 and miR-144) [207]. Additionally, *Lactiplantibacillus plantarum* PS128TM administration significantly improved cortisol levels, insomnia, and negative emotions [214]. Based on these studies, it can be inferred that probiotics have the potential to improve insomnia by regulating the HPA axis in both humans and animals. These findings suggest that probiotics can play a role in modulating the endocrine mechanisms involved in sleep regulation and stress response, thereby providing a potential therapeutic approach for insomnia.

Emerging research has highlighted the significant effects of various bacteria on the hypothalamic-pituitary-ovarian axis, which plays a role in regulating ovarian hormone metabolism and cerebral hormone secretion. (Fig. 4a). Jiachao et al. found that intervention with *Bifidobacterium animalis* subsp. *lactis* V9 stimulated the release of peptide YY and gastric hunger hormone by improving SCFA levels (acetic, propionic, and butyric acids) in the gut, and ultimately inhibited blood luteinising hormone and luteinising hormone/follicle stimulating hormone level [251]. β-glucuronidase being an enzyme responsible for deconjugating estrogen and allowing it to bind to estrogen receptors α and β [252,253]. A decrease in gut microbial diversity can negatively affect β-glucuronidase activity, leading to reduced estrogen levels [253], [254], [255]. This decrease is associated with increases in beneficial bacteria and inhibition of the growth of harmful bacteria [256]. Furthermore, there are interactions between the hypothalamic-pituitary-ovarian axis and many neurotransmitters For example, disruptions of the hypothalamic-pituitary-ovarian axis can result in reduced Mel secretion at night [34], and also effect 5-HT [257] and DA [258].

In summary, endocrine disorders involving cortisol and estrogen levels can contribute to insomnia. Probiotics and postbiotics have the potential to alleviate insomnia related to these endocrine disorders by regulating hormones, gut microbiota, and gut microbiota-derived metabolites.

### Immunomodulation

There is a close relationship between SRFs and the immune system, with various cytokines involved in sleep regulation present in the peripheral immune system (Fig. 4b). Notably, IL-1β can increase the release of NA and DA in the anterior hypothalamus and regulate the levels of 5-HT in the hypothalamus. TNF-α, on the other hand, can elevate the levels of 5-HT and its metabolite, 5-hydroxyindoleacetic acid, in the brain. Furthermore, TNF-α and IL-1β mainly regulate NREM sleep by influencing cortisol levels and activating the NF-κB pathway, which in turn leads to increased activity of the HPA axis [259]. Proinflammatory cytokines can also decrease the concentration of 5-HT by initiating the kynurenine pathway [260]. Lower levels of inflammatory factors promote sleep, improve sleep quality, and protect nerves. On the other hand, elevated levels of proinflammatory cytokines such as TNF-α, IL-6, and IL-1β can have a detrimental effect. These cytokines can heighten glutamatergic signaling while diminishing GABA signaling, resulting in heightened excitability of dorsal horn nociceptive cells and potential harm to host cells, including cells within the central nervous system [261]. Therefore, these proinflammatory cytokines serve as markers associated with insomnia, contributing to central sensitization and play a major role in SRF disorders involving sleep disturbances.

Furthermore, the immune system and the gut microbiota are intricately interconnected. Alterations in the gut microbiota and increased gut permeability are key contributors to systemic inflammation. For instance, the translocation of Gram-negative bacteria containing LPS leads to heightened levels of proinflammatory cytokines [262]. Consuming probiotics like SLAB51 could enhance the intestinal abundance of anti-inflammatory bacteria, such as *Bifidobacterium*, while reducing the concentration of pro-inflammatory taxa like *Campylobacterales*. Additionally, probiotics stimulate the growth of SCFA producers in the gut, resulting in elevated SCFA levels. The increase in anti-inflammatory and neuroprotective SCFAs in the intestine helps lower pro-inflammatory cytokine levels in the bloodstream and increase the concentrations of anti-inflammatory cytokines [263]. Moreover, probiotic supplementation has been shown to significantly reduce the levels of various pro-inflammatory cytokines, such as IL-12 and IL-4 [264], while considerably increasing levels of IL-10, glutamate, NO, and the total antioxidant capacity [265], [266], [267], [268], [269]. These findings highlight the anti-inflammatory potential of probiotics, through which insomnia is ameliorated.

### Neural regulation

The afferent vagus nerve serves as a neural pathway for communication between the gut and the brain, and it is also a key route through which probiotics can enhance brain function through the gut-brain axis. The vagus nerve connects the gut microbiota and symptoms related to insomnia. For example, mice underwent vagotomy or depleted of microbiota did not exhibit an inflammatory response related to SD [140,270], suggesting that both the gut microbiota and the vagus nerve are important players in the pathway underlying SD-related symptoms. Through this gut-brain axis, probiotics can improve brain function. Notably, the probiotic strain SBC8803 has been found to improve sleep diary scores and stimulate the vagus nerve [209]. The influence of SBC8803 on the vagus nerve can be counteracted by blocking 5-HT receptors. These observations lead to the hypothesis that SBC8803 impacts sleep through the vagal pathway, involving signaling mediated by 5-HT (Fig. 4c).

Furthermore, *Lacticaseibacillus rhamnosus* JB-1 has been found to reduce the transcription of GABA_A_ mRNA in the prefrontal cortex and amygdala, while increasing it in the hippocampus [271]. This suggests that probiotics can have varying effects on GABA in different brain regions. Moreover, studies have shown that intraduodenal injection of *Lactobacillus johnsonii* La1 increases the activity of efferent gastric vagus nerve, leading to increased food intake [272]. Conversely, *Lacticaseibacillus paracasei* ST11 has been found to inhibit the activity of efferent vagus neurons in rats, resulting in reduced food intake [273]. This suggests that probiotics exhibit strain-specific modulation of neurons, causing either initiation or inhibition. In conclusion, probiotics regulate insomnia through the vagus nerve, affecting neural pathways involving GABA and 5-HT, and their regulatory effects are strain-specific. Additionally, neurotoxic metabolites produced by the gut microbiota, such as D-lactic acid and ammonia, may enter the central nervous system through the vagus nerve, thereby influencing brain function [274].

Probiotics stabilize the HPA axis through the parasympathetic and vagus nerves, thereby maintaining the sleep-wake system. Intake of *Lacticaseibacillus casei* Shirota has been found to suppress the increase in corticosterone hormones, potentially improving sleep by signaling to the vagus nerve and reducing the stress response in the paraventricular nucleus [250]. These findings demonstrate that probiotics impact sleep through the vagus nerve pathway. However, further experiments are needed to characterize their strain-specific effects. By examining the expression of relevant receptors in the vagus nerve, the specific molecular mechanisms involved in insomnia improvement through the vagus nerve can be revealed.

It is worth mentioning that a recent study has revealed a correlation between gut protein concentrations of fruit flies and activity of gut endocrine cells. These cells release the peptide CCHa1, which sends signals to a small group of DA neurons in the brain, regulating the response of fruit flies to mechanical vibrations. Enhanced levels of CCHa1 enable fruit flies to enter sleep mode in response to more intense vibrations, while consumption of CCHa1 can induce wakefulness even in the presence of weak vibrations [275]. However, it is unclear whether probiotics and prebiotics have an effect on this signaling peptide and whether the peptide utilizes the vagus nerve to transmit its signals. In addition, whether there are other regulatory pathways for gut metabolites as well as the structure of the gut microbiota deserves further exploration (Fig. 4d). Meanwhile, we have also mentioned the relationship between mitochondria and sleep, and a large number of studies have demonstrated that mitochondrial function is closely related to gut microbiota [276]. However, the causal relationship between the two is still unclear. In addition to the pathway demonstrated in Fig. 4d, the mining of other pathways is also crucial. Therefore, subsequent analyses could focus on targeting induced impairment of mitochondrial function to validate its effects on sleep as well as between colonies.

The aforementioned findings provide compelling evidence that probiotics have the potential to ameliorate gut microbiota and metabolite disorders while utilizing neural, immune, and endocrine pathways to establish communication within the gut-brain axis. This intricate communication contributes to the alleviation of neuroinflammation, activation of the vagus nervous system, and regulation of brain function. Nonetheless, the current understanding of the mechanisms underlying probiotic interventions in sleep is still in its early stages. Sleep patterns are influenced by various factors, including alterations in gut permeability, immune system activity, inflammation, energy acquisition, and microbial diversity. The detailed pathways of how probiotics modulate the gut microbiota and the specific regulation involved in the gut-brain axis remain to be elucidated. In order to facilitate the reader 's interpretation, we added the abbreviation table at the end (Table 5).

**Table 5:** A list of abbreviations used in the study.

Abbreviation	Full Form	Abbreviation	Full Form
PCPA	4-Chloro-DL-phenylalanine	Mel	Melatonin
PG	Pineal gland	NO	Nitric oxide
5-HT	5-hydroxytryptamine	NOS	Nitric oxide synthase
ACh	Acetylcholine	NREM	Non-rapid eye movement
AD	Alzheimer's disease	NA	Noradrenaline
AANAT	Arylalkylamine N-acetyltransferase	ORX	Orexin
BF	Basal forebrain	REM	Rapid eye movement
BBB	Blood-brain barrier	SAMP8	Senescence-accelerated mouse prone-8
BDNF	Brain-derived neurotrophic factor	SCFAs	Short-chain fatty acids
DSM-5	Diagnostic and Statistical Manual of Mental Disorders, Fifth Edition	SD	Sleep deprivation
DA	Dopamine	SRFs	Sleep regulatory factors
ETC	Electron transport chain	SWS	Slow wave sleep
Erk	Extracellular signal-regulated kinase	TLR4	Toll-like receptor 4
Gal	Galanin	TST	Total sleep time
HA	Histamine	TMN	Tuberomammillary nucleus
HPA	Hypothalamic-pituitary-adrenal	TrkB	Tyrosine Kinase receptor B
LPS	Lipopolysaccharides	GABA	γ-aminobutyric acid
LC	Locus coeruleus	VLPO	Ventrolateral preoptic nucleus
MCH	Melanin concentration hormone		

## Conclusions

Recent advancements have been made to understanding the connection between sleep and gut microbiota. Insomnia may lead to physiological and psychological changes that are closely intertwined with the gut microbiota, including metabolic disorders, inflammatory reactions, and emotional disturbances arising from insufficient sleep. From the perspective of the gut-brain axis, it has been established that impaired brain functions due to insomnia can adversely affect the gut function and microbiota, making probiotics a viable alternative to prescription medication for insomnia. However, further research is needed to establish the causal relationship between compromised intestinal function, gut dysbiosis, and insomnia symptoms.

Administrating probiotics and postbiotics has emerged as an effective method of restoring a healthy gut microbiota, thereby improving disorders and metabolic disturbances associated with gut dysbiosis. Mechanisms in relation to probiotics for sleep improvement are rooted in the gut-brain axis, alterations in gut microbiota associated with insomnia, inflammatory responses, and the neuro-modulatory mechanisms of sleep. Probiotics can regulate sleep-wake behavior by modulating gut microbiota and its metabolites, influencing endocrine, neuronal, and immune responses. However, currently, the mechanism by which probiotics improve insomnia has not been extensively validated, primarily due to the lack of a suitable animal model.

To understand the role of probiotics in improving insomnia, a standardized sleep behavior index and sleep monitoring method are needed. Enhancements to the insomnia animal model are also necessary to make it more accurate and comparable to human insomnia. Most importantly, the experimental design should be sound, with experiments conducted on insomnia models induced by different drugs and interventions involving sleep medications and probiotics. Analyzing and comparing the mechanisms of action between probiotics and sleep medications can help elucidate the probiotic mechanism at a deeper level. The implementation of interdisciplinary methods like the emerging single-cell spatial multi-omics techniques would enable detailed investigation of gene expression, metabolism, and cell arrangement in tissues with subcellular resolution. Recent studies have utilized these techniques to analyze gene expression in various regions of the mouse brain and spinal cord, providing precise spatial gene expression profiles [280]. Additionally, high-resolution cell maps have been constructed to study age-related gene expression changes in the frontal cortex and striatum of mice [280,281]. Interestingly, non-neuronal cells exhibited more pronounced age-related alterations compared to neurons, with distinct spatial patterns. Applying this technology to study brain gene expression profiles during insomnia may offer novel insights into the underlying mechanisms of this sleep disorder.

Ongoing clinical research on insomnia offers the opportunity to explore the impact of probiotics on sleep from multiple perspectives. Over all, the analyses show that probiotics modulate insomnia as well as insomnia-induced inflammation, gut barriers and other types of adverse effects by regulating gut microbiota and metabolites that affect substances such as SRFs and cytokines. These findings not only improve our understanding of probiotic regulation of insomnia, but also provide new perspectives for clinical treatment. In particular, probiotics have the potential to improve patient prognosis, which may have a profound impact on clinical decision making. Additionally, the study of insomnia mechanisms is intricate, necessitating a solid foundation in basic research. Gaining a deeper understanding sleep structure and patterns, delving into specific populations, such as perimenopausal women with insomnia, and leveraging sophisticated technologies like single-cell spatial multi-omics can provide insights into how probiotics influence the metabolic expression of specialized cells or tissues before and after intervention. This knowledge has the potential to support tailored microbial psychiatric treatment strategies. To achieve clinical translation of these findings, future research needs to explore the synergistic effects of probiotics with other dietary supplements on insomnia. We also recommend using a combination of subjective and objective methods to fully assess sleep quality. We recommend that clinicians and researchers closely monitor advances in this area and consider integrating this evidence into clinical practice. Through these efforts, we can expect to see more effective clinical management and improved patient outcomes in the area of insomnia.

## Compliance with ethics requirements

This article does not contain any studies with human or animal subjects.

## CRediT authorship contribution statement

**Qiong Wu:** Writing – original draft. **Guangqi Gao:** Writing – original draft. **Lai-yu Kwok:** Writing – original draft. **Huimin Lv:** Visualization. **Zhihong Sun:** Conceptualization, Project administration.

## Funding

We are profoundly thankful to the 10.13039/501100001809National Natural Science Foundation of China ([32325040), the 10.13039/501100012166National Key Research and Development Program of China ([2022YFD2100702), Inner Mongolia Agricultural University First-Class Discipline Scientific Research Special Program (YLXKZX-NND-006), Research Initiation Project for Introducing Excellent Doctoral Talents from Inner Mongolia Agricultural University (NDYB2022-41) and the earmarked fund for CARS36.

## Declaration of competing interest

The authors declare that they have no known competing financial interests or personal relationships that could have appeared to influence the work reported in this paper.
